# Reductive diazotation of carbon nanotubes: an experimental and theoretical selectivity study†
†Electronic supplementary information (ESI) available: Experimental details regarding the reductive charging of HiPco SWCNTs and additional Raman and TGA analysis. In addition, theoretical details regarding the structural parameters, the calculated structures of the SWCNT/diazonium interaction, and the electronic properties of the studied system. See DOI: 10.1039/c8sc03737j


**DOI:** 10.1039/c8sc03737j

**Published:** 2018-10-22

**Authors:** Milan Schirowski, Christoph Tyborski, Janina Maultzsch, Frank Hauke, Andreas Hirsch, Jakub Goclon

**Affiliations:** a Chair of Organic Chemistry II & Joint Institute of Advanced Materials and Processes , Friedrich-Alexander University of Erlangen-Nürnberg , Nikolaus-Fiebiger-Straße 10 , 91058 Erlangen , Germany . Email: andreas.hirsch@fau.de; b Institut für Festkörperphysik , Technische Universität Berlin , Hardenbergstraße 36 , 10623 Berlin , Germany; c Chair of Experimental Physics , Friedrich-Alexander University Erlangen-Nürnberg , Staudtstr. 7 , 91058 Erlangen , Germany; d Institute of Chemistry , University of Bialystok , Ciolkowskiego Str. 1K , 15-245 Bialystok , Poland . Email: j.goclon@uwb.edu.pl

## Abstract

The reaction of neutral single-walled carbon nanotubes (SWCNTs) with diazonium salts proceeds with a high selectivity towards metallic carbon nanotube species; this reaction is well-understood and the mechanism has been elucidated. In the present joint theoretical and experimental study, we investigate the reaction of negatively charged SWCNTs – carbon nanotubides – with diazonium salts. Our density functional theory calculations predict a stronger binding of the aryl diazonium cations to charged metallic SWCNTs species and therefore lead to a preferential addend binding in the course of the reaction. The Raman resonance profile analysis on the reductive arylation of carbon nanotubides obtained by the solid state intercalation approach with potassium in varying concentrations confirms the predicted preferred functionalization of metallic carbon nanotubes. Furthermore, we were also able to show that the selectivity for metallic SWCNT species could be further increased when low potassium concentrations (K : C < 1 : 200) are used for an initial selective charging of the metallic species. Further insights into the nature of the bound addends were obtained by coupled thermogravimetric analysis of the functionalized samples.

## Abstract 2

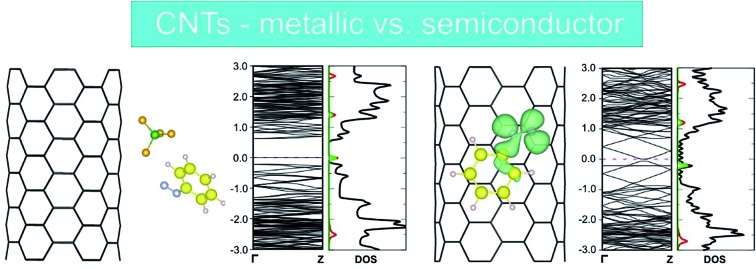
The reaction of negatively charged SWCNTs with diazonium salts was analyzed in a combined experimental and computational DFT study.

## Introduction

The field of single-walled carbon nanotube (SWCNT) chemistry has steadily developed during recent years and has become a very important subject within the field of carbon allotrope research.^1^–^3^ The covalent attachment of functional entities allows for the adjustment of their dispersibility,^4^ the tuning of their electronic properties for applications in batteries or supercapacitors,^5^ or for the introduction of anchoring groups to enable subsequent coupling reactions of highly versatile building blocks.^6^–^9^ However, for their vast implementation in high-performance components, a number of substantial hurdles have still to be overcome. One major challenge is the efficient and high-yield separation of either metallic or semiconducting carbon nanotubes, as SWCNT bulk synthesis always yields mixtures of tubes with both electronic types as well as different diameters and lengths. In a ground-breaking work, Strano *et al.*^10^ have shown that the covalent functionalization of surfactant-individualized, neutral SWCNTs with diazonium salts proceeds with a high selectivity towards metallic carbon nanotube species. On this basis, SWCNTs can be sorted by metallicity utilizing electrophoresis^11^ or density gradient methods.^12^,^13^ A fundamental prerequisite for this electronic type selective functionalization is a successful individualization of the neutral carbon nanotube species by application of highly efficient surfactant molecules and meanwhile, the reaction is well-understood and the mechanism has been elucidated.^14^,^15^ An alternative route to individualized SWCNT is based on reductive charging of the carbon nanotubes^16^–^18^ resulting in electrostatic repulsion of the respective negatively charged intermediates, the so-called nanotubides.^19^ The SWCNT reduction can be accomplished *via* Birch type protocols,^17^,^18^ with organic electron transfer reagents like naphthalene,^20^–^22^ or as we have recently demonstrated by direct application of alkali metals like potassium.^23^,^24^ Beyond charge-driven dissolution, the raised Fermi level of reductively charged SWCNTs can be used to activate a broad variety of suitable electrophilic trapping reagents in the course of a single electron transfer reaction.^25^ This leads to the formation of alkyl or aryl radicals, which subsequently are highly efficiently coupled covalently to the carbon nanotube sidewall.^18^,^26^ For the reductive functionalization protocol alkyl- and aryl halides,^27^ λ^3^-iodanes, and also diazonium cations can be utilized. With respect to the electronic type specific functionalization of reductively charged carbon nanotubes we were able to show that the reaction of carbon nanotubides generated *via* a Birch type protocol with benzenediazonium tetrafluoroborate proceeds in a fashion where metallic carbon nanotubes were predominantly functionalized by the intermediately generated aryl radicals. This observation directly leads to the question which parameters are responsible for an electronic type selective functionalization of carbon nanotubes in the course of a reductive alkylation/arylation scenario and this question has up to now not fully been addressed by theory. The problem with a Birch type scenario is that many different parameters (like the amount of negative charges applied towards the SWCNTs, electride formation and the presence of liquid ammonia)^28^ are involved in this approach, which could potentially be part of the selectivity-determining step and their influence could not exactly be controlled and deconvolved.

In order to elucidate the underlying driving forces for an electronic type selective reaction of carbon nanotubides we reduced the amount of parameters by charging the SWCNTs with the alkali metal potassium in a clean and controllable solid state reaction.^23^,^24^ The alkali metal concentration can be varied and has a direct influence on the final degree of functionalization. Moreover, in a previous joint experimental and theoretical investigation, we were able to show that for low K : C ratios of <1 : 200 the adsorption of a potassium atom on metallic carbon nanotubes is always preferred over the adsorption on semiconducting tubes, independently of the tube diameter.^29^ In principle, this selective charging of metallic carbon nanotubes in the low charge density regime can be exploited for a charge-driven separation approach. On the other hand, in typical functionalization experiments^27^ vastly higher potassium : carbon charging ratios are used and therefore any observed electronic type selective arylation and alkylation of nanotubide intermediates cannot simply be referred to a selective charging of specific helicities in the starting mixture.

**Scheme 1:**
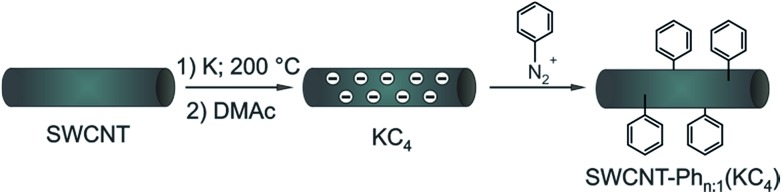
Arylation of reductively exfoliated carbon nanotubides, obtained by a solid state intercalation process with potassium (K : C ratio of 1 : 4) by electrophilic trapping with benzenediazonium cations. Three samples SWCNT–Ph_*n*:1_(KC_4_) (*n* = 1, 4, 20) were synthesized with varying diazonium : nanotube carbon ratios (1 : 1, 4 : 1, and 20 : 1) – see also Table 2.

Identifying the selectivity-determining step of the reaction is a challenging task which can hardly be solved by an experimental approach only. Consequently, in order to understand the underlying principles of electronic type selective SWCNT reactions, we focused in our present study on the well-defined reaction of a diazonium cation with solid state charged carbon nanotubes. The advantage of this protocol is the absence of further co-reducing agents such as liquid ammonia or naphthalene, which would severely obstruct a comparison of the experimental data with the theoretical predictions. Based on a density functional theory (DFT) approach, in the present work we investigated the direct interaction of the electrophilic trapping reagent with the negatively charged carbon nanotubides of both electronic types. We could verify that aryl diazonium cations bind stronger to charged metallic species than to semiconducting ones. Moreover, a higher density of states of SWCNTs in the vicinity of the singly occupied molecular orbital/lowest occupied molecular orbital (SOMO/LUMO) of the aryl diazonium radical/cation pair – *i.e.* higher charging density – directly leads to a more efficient electron charge transfer from the charged nanotubides to the adsorbed electrophilic trapping reagent.^13^ In addition, the calculations reveal no correlation of the PhN_2_ radical/cation-SWCNTs interaction energy with varying tube diameters. Based on these theoretical results, charged metallic SWCNTs should be more reactive than their semiconducting counterparts, which is in perfect agreement with the results we obtained on Birch type reduced systems.^27^ In the present experimental approach, we carried out the SWCNT reduction in a well-defined potassium intercalation^30^ protocol with a variation of the applied charge density in the course of the reductive diazotation reaction (Scheme 1).^29^ The successful arylation of the carbon nanotubes was verified by a thermogravimetric (TG) product analysis, coupled with gas-chromatographic (GC) separation and mass-spectrometric (MS) characterization (TG-MS and TG-GC-MS). Moreover, by in-depth Raman analysis of the RBM (radial breathing mode) region of the covalently functionalized carbon nanotubes, we could prove that the reductive diazotation leads to a preferred functionalization of metallic SWCNT species, as predicted by our DFT calculations, and that at potassium concentrations below the 1 : 200 threshold the selectivity for metallic carbon nanotubes could be increased in a cooperative utilization of two selectivity-determining parameters.

## Computational and experimental details

### DFT calculations

The computational results presented in this study were obtained using the PWscf program^31^ based on density functional theory (DFT). The Kohn–Sham wave functions were expanded in plane waves up to a cutoff of 30 Ry. Core electrons were replaced by Vanderbilt ultrasoft pseudopotentials.^32^ The generalized gradient approximation (GGA) using Perdew–Burke–Ernzerhof (PBE) functional^33^ with Grimme's correction (D2) was used to describe van der Waals interactions.^34^ The total energy minimization was performed by using Hellman–Feynman forces. All structures were fully relaxed until the forces acting on atoms were less than 11 meV Å^–1^. Periodic boundary conditions were applied to obtain an infinite nanotube length. The starting geometries of the nanotubes were generated with the Tube-Gen 3.4 web interface.^35^ All carbon nanotubes were initially optimized and taken to investigate the interactions with aryl diazonium cations. To study the negatively charged SWCNTs as well as positively charged aryl diazonium cation a compensating jellium background charge density was added to periodic boundary conditions. For each case, one extra electron was added to the SWCNT supercell due to the fact that we have studied the interactions with one aryl diazonium cation. The vacuum space in the lateral directions was set to be larger than 14 Å in order to eliminate the interactions between the periodic images. The simulated pristine SWCNTs consisted of 160–532 carbon atoms and had lengths in the range of 17.14–49.26 Å. The supercell representing (10,0) and (6,6) SWCNT contained four unit cells, the supercell representing (12,3) tube contained three unit cells, while all other supercells were formed from one unit cell. For the Brillouin zone integration along the tube axis (*z*-direction), a Monkhorst–Pack sampling scheme^36^ was adopted. The *k*-point sampling and detailed geometrical parameters (optimized length of the supercell, diameter) for all studied pristine SWCNTs are listed in Table 1. A *k*-point mesh of 1 × 1 × 10 was used in the case of density of states (DOS) calculations with a Gaussian broadening of 0.05 eV for the energy eigenvalues. The figures were made using VESTA^37^ and XCrySDen^38^ programs.

**Table 1:** Structural parameters for optimized SWCNTs using the DFT(PBE+D2) approach. The presented parameters contain number of carbon atoms (*n*), optimized length of the supercell (*l*_opt_), diameter (*d*), and corresponding *k*-point sampling for integration in the first Brillouin zone

SWCNT	*n*	*l*_opt_/Å	*d*/Å	*k*-point
(10,0)	160	17.14	7.89	1 × 1 × 4
(9,4)	532	49.26	9.06	1 × 1 × 1
(7,5)	436	44.61	8.25	1 × 1 × 1
(8,3)	388	42.07	7.75	1 × 1 × 1
(6,6)	168	17.27	8.21	1 × 1 × 4
(13,4)	316	21.94	12.08	1 × 1 × 2
(13,1)	244	19.26	10.66	1 × 1 × 2
(12,3)	252	19.60	10.84	1 × 1 × 2

### Materials

HiPco SWCNTs (grade: pure; lot number P2772, TGA residue 15 wt%, diameter 0.8–1.4 nm, length 100–1000 nm) were obtained from Unidym and used without further treatment. Dimethylacetamide (DMAc)^20^ was used as solvent and purified according to the procedure outlined below. All other chemicals were purchased from Sigma-Aldrich and used without further treatment if not stated otherwise.

### Solvent preparation

HPLC-grade DMAc was dried over molecular sieve (3 Å, 15%/w) for 3 days and the remaining water content of <10 ppm was confirmed by Karl-Fischer titration. Subsequently, the solvent was deoxygenated *via* freeze–pump–thaw procedure (four cycles).

### SWCNT charging, dispersion, functionalization: SWCNT–Ph_C:Ph_(KC_*x*_)

Sample functionalization was carried out in an argon-filled Labmasterpro sp glovebox (MBraun), equipped with a gas purifier and solvent vapor removal unit, with both oxygen and water content <0.1 ppm. In the denotation, the subscripted numbers represent the molar ratio of nanotube carbon to benzene diazonium, and – in brackets – the molar ratio of potassium to nanotube carbon, respectively.

#### Synthesis of SWCNT–Ph_(4:1)_(KC_4_)

6.0 mg (0.50 mmol) of HiPco SWCNTs were pre-dried *in vacuo* and loaded into the glove box. In a 20 mL glass vial, the SWCNTs were charged with 4.9 mg (0.13 mmol) potassium and stirred occasionally with a metal spatula at 200 °C. After 24 h, the bronze-colored carbon nanotubide salt was dissolved in 6 mL of DMAc (without any sonication) and a homogenous black dispersion was formed. 19.2 mg of benzenediazonium tetrafluoroborate (0.13 mmol) was added and the dispersion was stirred for 24 h. Subsequently, the reaction mixture was unloaded from the glove box and exposed to air. The material was filtered through a 0.25 μm reinforced cellulose membrane filter (Sartorius) and washed with 100 mL acetone and water, each. The resulting black powder was dried *in vacuo*.

The other functionalized SWCNT–Ph_C:Ph_(KC_*x*_) samples were synthesized *via* the same procedure, only altering the molar amounts of potassium and diazonium salt according to Table 2.

**Table 2:** Variation of the amount of potassium and trapping electrophile for the synthesis of the respective SWCNT–Ph_C:Ph_(KC_*x*_) derivatives, with the resulting potassium : diazonium ratio. Nanotube carbon amount was constant at 0.50 mmol

Sample	*n* (K)/mmol	*n* (diaz.)/mmol	Ratio K : diazonium
SWCNT–Ph_(1:1)_(KC_4_)	0.13	0.50	1 : 4
SWCNT–Ph_(4:1)_(KC_4_)	0.13	0.13	1 : 1
SWCNT–Ph_(20:1)_(KC_4_)	0.13	0.025	5 : 1
SWCNT–Ph_(10:1)_(KC_200_)	0.0025	0.050	1 : 20

### Sample characterization

Statistical Raman characterization was carried out on a Horiba Jobin Yvon LabRAM Aramis confocal Raman microscope (excitation energy: 2.33 eV & 1.58 eV) with a laser spot size of ∼1 μm (Olympus LMPlanFl 100×, NA 0.80) in backscattering geometry. A silicon detector array charge-coupled device (CCD) at –70 °C was used to record the spectrum. The spectrometer was calibrated in frequency using crystalline graphite prior to measurement. Statistical Raman measurements were obtained through a motorized *x*–*y* table in a continuous linescan mode (SWIFT-module) in order to guarantee fast data acquisition. Maps of 100 μm × 100 μm were recorded with a step size of 4 μm, yielding a total of 626 single-point spectra with acquisition times of 0.5 s each. Laser power was kept <0.6 mW to avoid defunctionalization of the nanomaterial. Displayed spectra depict the mean of 626 spectra and *I*_D_/*I*_G_ ratios were determined by statistical evaluation.^27^

Raman measurements for the in-detail screening of the RBM modes were performed in backscattering geometry under standard ambient conditions. An Ar^+^/Kr^+^ and an Ti : Sa laser were used to tune the excitation energies between 1.50 eV and 2.73 eV. Laser power was kept below 0.6 mW at all times. The tunable laser setup allowed to probe metallic M11, as well as semiconducting S22, S33, and S44 electronic transitions with diameters between ∼7 and 13 Å. Raman spectra were taken with a Dilor XY800 triple monochromator and a neon lamp was used for frequency calibration. A final assignment of SWCNTs in the sample was carried out *via* pattern recognition.^39^–^41^ For further details, a full characterization of another HiPco sample can be found in the literature.^39^,^42^

Thermogravimetric analysis coupled with mass spectrometry: TG-MS (online) was performed on a Netzsch Skimmer STA 409 CD instrument with a quadrupole in EI^+^ mode. Samples were heated with a constant rate of 20 K min^–1^ from room temperature to 700 °C under a constant flow of helium (80 mL min^–1^).

Thermogravimetric analysis coupled with gas chromatography and mass spectrometry: TG-GC-MS was performed on a Pyris 1 TGA from Perkin-Elmer coupled with a Clarus 680 gas chromatograph with an Elite-5 column and an SQ 8 quadrupole mass spectrometer. Samples (2–4 mg) were heated with a constant rate of 20 K min^–1^ from room temperature to 700 °C. Helium with a constant flow of 1 mL min^–1^ was used as carrier gas. Injection for the detection of covalently attached aryl moieties was performed at 280 °C, for the decomposition products of SWCNT–Ph_(20:1)_(KC_4_) at 480 °C (temperatures determined by TG-MS results). The GC temperature ramp was 40–280 °C with a constant heating rate of 10 K min^–1^ and a final 10 min plateau at the maximum temperature. The obtained data was processed with TurboMass software and bibliographic searches where carried out with NIST MS Search 2.0.

## Results and discussion

**Fig. 1:**
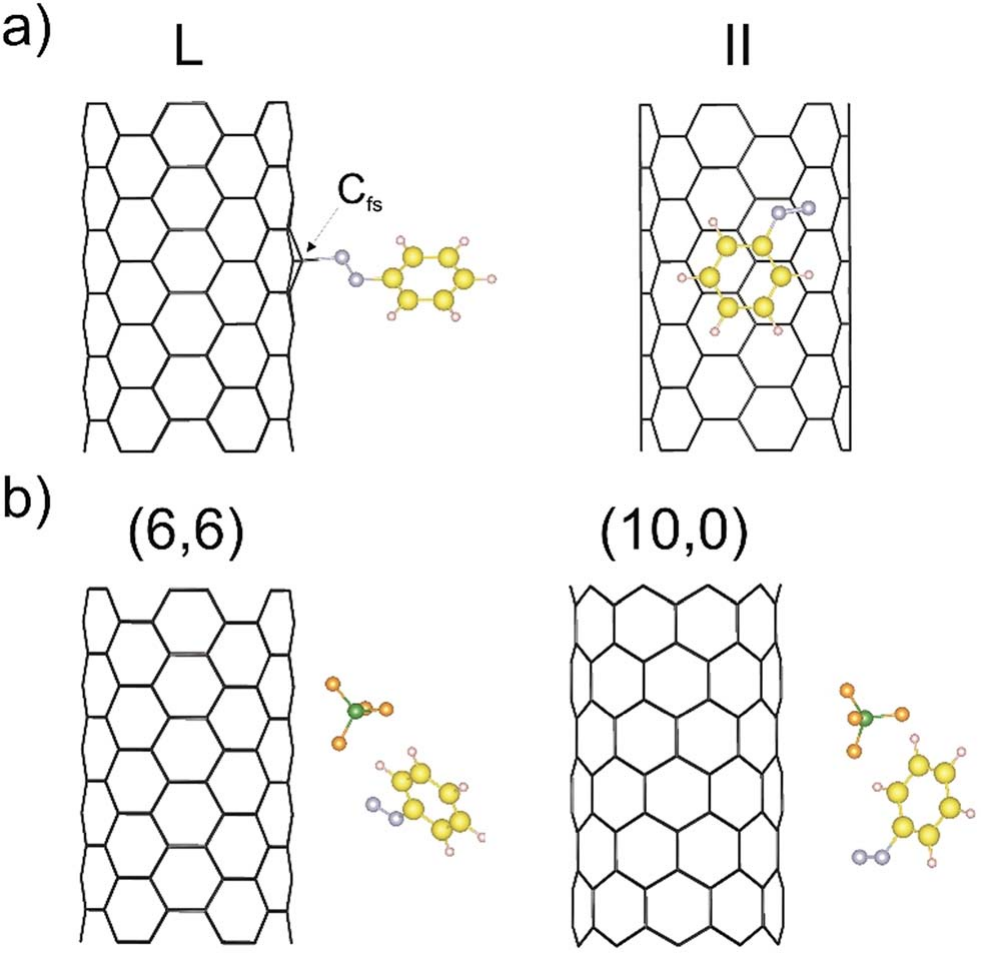
(a) The perpendicular (L) and parallel (II) interaction types of the benzenediazonium cation with the charged m-(6,6) SWCNT^–^ after geometry optimization. C_fs_ denotes the carbon atom at the functionalization site. (b) Optimized geometry for the m-(6,6)/sc-(10,0)–PhN_2_^+^ conjugate (L configuration) after including the BF_4_^–^ counter anion.

As outlined in the introduction, we have already obtained some experimental results which indicate that the reductive diazotation of negatively charged carbon nanotubides proceeds in an electronic type selective reaction, where metallic carbon nanotube species are predominantly arylated. To identify the selectivity-determining step we took a closer look at the direct interaction of the diazonium cation with the charged SWCNTs of both electronic types, metallic and semiconducting, by a DFT approach. In order to reproduce the experimental conditions as closely as possible, we have introduced to our theoretical model negatively charged SWCNTs of different helicities and diameters. Our theoretical investigation is based on eight SWCNT chiral indices – (10,0), (8,3), (7,5), (9,4), (6,6), (12,3), (13,1), and (13,4) – where the first four are of semiconducting (sc) type and the latter four are metallic (m). This representative choice is motivated by several reasons. First of all, the pairs sc-(10,0) and m-(6,6) have comparable diameters of 7.89 and 8.21 Å, respectively, and, as achiral tubes, represent systems with the lowest number of atoms per unit cell,^43^ reducing the computational load (Table 1). Therefore, the direct comparison of the SWCNT^–^/diazonium^+^ interaction with respect to the differences between metallic and semiconducting species has been carried out with this pair of SWCNT helicities. Secondly, the other selected tubes cover a diameter range of 8.3–12.1 Å – the range of the majority of tube diameters that are present in standard HiPco SWCNTs – with sc-(7,5) and sc-(9,4) as the most abundant species.^9^,^44^ Firstly, the position of one aryl diazonium cation on the sidewall of metallic and semiconducting carbon nanotubides was studied. Two initial configurations were considered: where the plane of the aryl group was perpendicular (L) or parallel (II) to the SWCNT axis. Fig. 1a depicts two possible structures of the m-(6,6)–PhN_2_ conjugate (systems with other chirality are presented in Fig. S1†). The perpendicular arrangement (L) of the aryl group is connected with the C_fs_-N bond formation between the nitrogen atom from the cation and the carbon atom at the functionalization site (C_fs_). The corresponding bond lengths for all studied systems are in the range from 1.56 to 1.64 Å, see Table 3.

**Table 3:** Calculated binding energy values and distances. The binding energy values, *E*_b_ [eV] and selected distances [Å] for perpendicular (L) and parallel (II) arrangement of the benzenediazonium cation (PhN_2_^+^) on different negatively charged SWCNTs were calculated on the basis of a DFT(PBE)+D2 approach. The C_fs_-N denotes the distance between the nitrogen atom from the attached cation and the carbon atom (marked as C_fs_) from the nanotube at the functionalization site. C···C refers to the distance between all carbon atoms from the aryl group and the closest carbon atom from the SWCNT. Semiconducting tubes given in italics, metallic SWCNT types given in bold

SWCNT type	C_fs_–N [Å]	C···C [Å]	*E*_b_ [eV]	
	L	II	L	II
*(10,0)*	1.58	3.28–3.50	2.77	3.61
*(9,4)*	1.59	3.27–3.49	2.55	3.53
*(7,5)*	1.59	3.26–3.52	2.53	3.44
*(8,3)*	1.58	3.37–3.58	2.60	3.44
**(6,6)**	1.56	3.28–3.51	2.71	3.45
**(13,4)**	1.64	3.19–3.55	2.88	3.97
**(13,1)**	1.60	3.27–3.55	2.80	3.80
**(12,3)**	1.60	3.10–3.43	2.70	3.33

The parallel arrangement (II) of the benzenediazonium cation and the charged SWCNTs originates from van der Waals type mutual interactions. The initial PhN_2_^+^ has been transformed into the radical with the N–N bond distances and the N–N–C angles of 1.19 Å and 125.5°, respectively. The obtained structural parameters are similar to those obtained for the radical structure in the gas phase (see Fig. S3†). The shortest C–C distances between the aryl group and the SWCNT are in the range of 3.10 to 3.58 Å (see Table 3), which suggests the dominant role of the π–π stacking interactions. This is in agreement with the results of the DFT(PBE)+D2 calculations for non-covalently functionalized SWCNTs with other aromatic molecules.^45^

Apart from the structural parameters, the reactivity of the systems was also analyzed in terms of its energetic properties. The binding energy (*E*_b_) between the benzenediazonium cation (PhN_2_^+^) and the charged nanotubide (SWCNT^–^) can be quantified by eqn (1):

1 *E*_b_ = (*E*_SWCNT^–^_ + *E*_PhN_2_^+^_) – *E*_SWCNT–PhN_2__

where *E*_SWCNT–PhN_2__ (*E*_SWCNT^–^_) is the total energy of the system (charged SWCNT) and *E*_PhN_2_^+^_ denotes the total energy of the isolated cation. All values of binding energy for the two types of arrangements (II, L) are summarized in Table 3.

The binding energies (*E*_b_) are very high because of the unbalanced charge of the individual components, therefore, only the relative values should be compared. Since the SWCNTs^–^ anion with attached PhN_2_^+^ cation contain an odd number of electrons in the supercell, both spin-polarized and spin-unpolarized schemes were included in the computational setup. For the perpendicular arrangements (L) the singlet and doublet state yields almost the same binding energy, whereas in the parallel case (II) the doublet state is always energetically favourable, unambiguously showing the formation of the PhN_2_ radical on the nanotube sidewalls. However, it may happen that the energy difference between the singlet and the doublet state is small, like for the m-(12,3)–PhN_2_ (II) arrangement, namely –0.03 eV. Such small energy difference makes the discussion about the stability of the system hard due to the limited accuracy of the DFT. Here, the analysis of the structural parameters of the attached PhN_2_ group shows its similarity to the structure of PhN_2_^+^ in the gas phase.

The computed binding energies (*E*_b_) are on average higher for both types of the arrangement for the metallic nanotubes, with the maximum differences of about 0.3–0.5 eV. The results obtained by Sumpter *et al.*^46^ for interactions between the aryl diazonium cation and neutral SWCNTs for the perpendicular (L) arrangement also show the enhanced stability for the interaction with metallic SWCNT species. In such cases, the increase of the C_fs_-N bond length with increasing nanotube diameter is observed (see Tables 1 and 3). Regardless of the diameters, the parallel arrangement (II) is energetically favored by 0.7–1.1 eV in comparison to the perpendicular (L) one. The binding energies (*E*_b_) obtained do not depend on the nanotube diameter but rather on the chirality type. This is similar to the results obtained by Umeyama *et al.*^47^ for the attachment of a single 4-(1-pyrenyl)phenyl radical on the SWCNTs sidewalls of various chiralities. Furthermore, it has been shown^46^ that if the diazonium salt anion (BF_4_^–^) is included in the calculations, there is a large preference for PhN_2_^+^ to associate with it, instead of being deposited on the surface of the carbon nanotube. Taking into account the presence of the BF_4_^–^ anion in the smallest conjugates, namely sc-(10,0)/m-(6,6)–PhN_2_, with the perpendicular arrangement (L), the formation of the PhN_2_···BF_4_^–^ complex on the nanotube sidewall (*E*_b_ of 2.61/2.39 eV) is observed (Fig. 1b). Therefore, we can conclude that the diazonium salts interact primarily with SWCNTs *via* van der Waals interactions before the N_2_ is released and the aryl radical is formed.

**Fig. 2:**
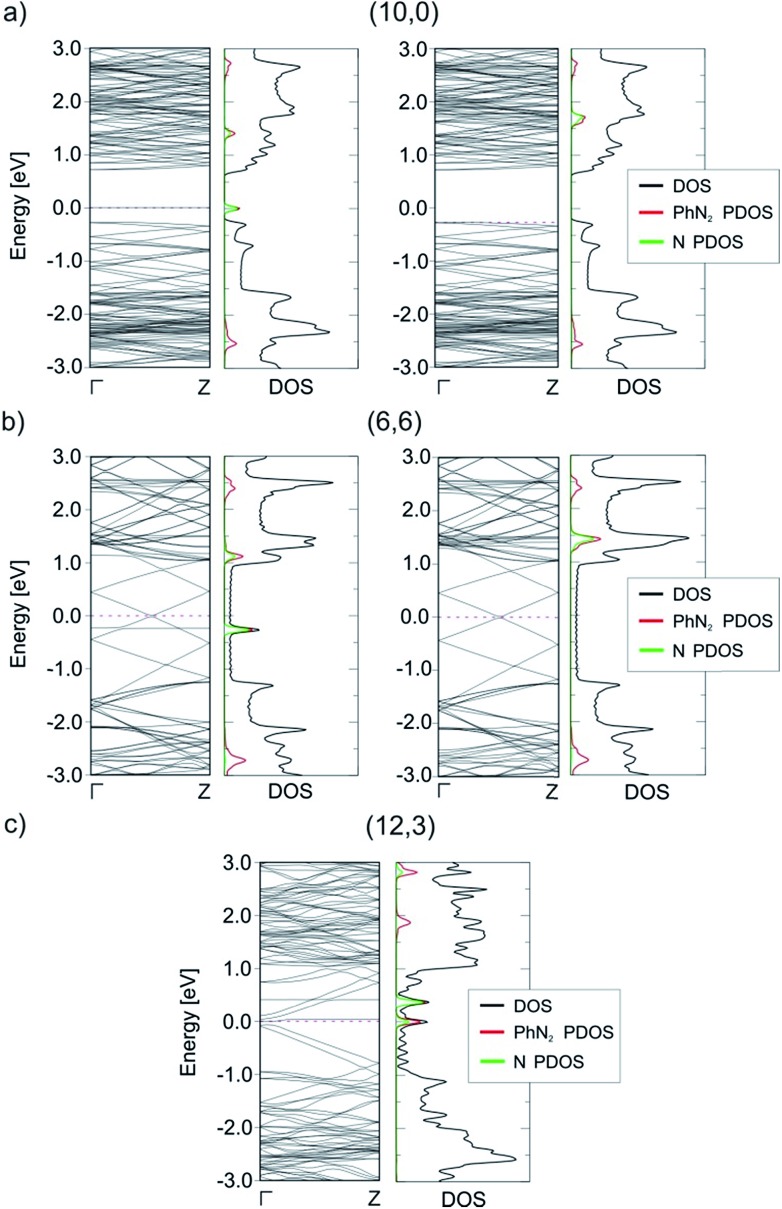
Band structures and total and projected (*P*) density of states (DOS) of the sc-(10,0)/m-(6,6)/m-(12,3)–PhN_2_ conjugates with the parallel (II) arrangement. For the spin-polarized systems, the left side of the graph corresponds to the spin-up, the right side to the spin-down electronic contribution. The pink dashed lines correspond to the maximally occupied spin-up and spin-down channels. The Fermi energy is set to 0 eV.

**Fig. 3:**
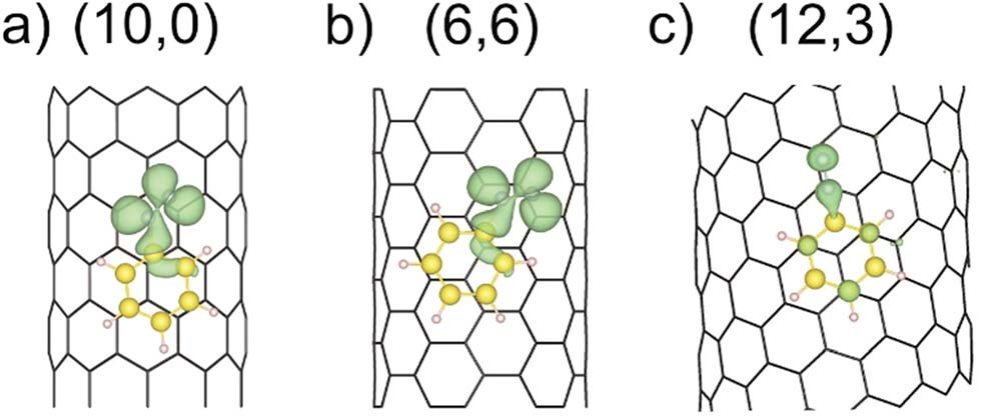
The electron density of the Γ-point wave function of the SOMO (a and b) and LUMO (c) of the aryl diazonium radical/cation for three different cases, see Fig. 2. The green surface represents an isovalue of 2 × 10^–3^ Bohr Å^–3^.

The reduction of the diazonium cation in the course of an electron transfer reaction and subsequent radical formation is the rate-limiting step. Electron transfer occurs between any occupied state of the SWCNTs that matches in energy with an unoccupied state of the benzenediazonium cation. Such studies have previously been carried out for the functionalization of graphene with diazonium salts.^48^,^49^ By merging the electronic band structure with the chemical molecular orbital theory, the different reactivities of metallic and semiconducting SWCNTs can be explained. Fig. 2 shows the electronic structure (band structure and DOS) for the sc-(10,0)/m-(6,6)–PhN_2_ (II) conjugates. The results for the other SWCNT helicities under investigation are summarized in the ESI (Fig. S2).† The (6,6) carbon nanotube exhibits a metallic character (with a zero band gap) while the (10,0) tubes represent semiconducting species with a band gap of 0.8 eV. Significant differences in the DOS near the Fermi level are observed for the semiconducting and metallic systems. In the case of sc-SWCNTs–PhN_2_ conjugates, the appearance of a half-occupied non-dispersive band in the energy gap of semiconducting nanotubes is clearly seen. Fig. 3a presents the electron density of the Γ-point wave function of this band. This band corresponds to the singly occupied molecular orbital (SOMO) of the PhN_2_ radical that is mainly composed of the nitrogen orbitals (PDOS) (for details see Fig. S3†). The SOMO level always lies some distance from the top of the valence band of the sc-SWCNTs. Therefore, there is no overlap with the electronic states of the nanotube. In the case of the m-SWCNT–PhN_2_ conjugates, the SOMO is located about 0.25 eV below the Fermi level (see Fig. 2b and S3a†). As a result, the electron transfer should easily proceed between the charged m-SWCNT and the adsorbed cation. For the m-(12,3)–PhN_2_ conjugate (singlet state) a different electronic structure is observed (Fig. 3c). The attached PhN_2_ molecule retains the initial electronic configuration of the cation (the N–N bond distance of 1.13 Å and the N–N–C angle of 180.0°) where the lowest unoccupied molecular orbital (LUMO) (Fig. 3c and S3b†) is only partially occupied at the Fermi level. The common feature of the studied SWCNT–PhN_2_ systems is that metallic nanotubes have higher density of states (DOS) in the vicinity of the SOMO/LUMO of the benzenediazonium radical/cation.

From the experimental side, the electronic type selective arylation of intermediately charged carbon nanotubides has previously been reported for a Birch type reduction protocol with benzenediazonium tetrafluoroborate as trapping reagent.^27^ In this functionalization scenario, metallic carbon nanotubes were predominantly functionalized by the intermediately generated aryl radicals. In the present joint experimental and density-functional theory (DFT) study we investigated if the results obtained for the reductive diazotation under Birch type conditions can be transferred to other SWCNT reduction protocols. Simultaneously, we shed light on the underlying principles which determine the electronic type selectivity of the functionalization of carbon nanotubides with diazonium cations. Here, we focused on the reductive solid-state intercalation/charging of carbon nanotubes by potassium (Scheme 1) as the variation of the amount of reducing agent within the reductive activation step is one fundamental parameter to adjust the degree of functionalization in the respective SWCNT derivatives.^23^ The advantage of this charging protocol^23^,^24^,^50^ over alternative nanotubide generating approaches is the absence of further co-reducing agents such as liquid ammonia or naphthalene.^17^,^20^,^25^

Moreover, this solid state SWCNT activation protocol inherently allows to adjust the stoichiometric amounts of the reducing agent as well as that of the trapping electrophile over a wide range with high accuracy. For the generation of individualized, charged, and highly reactive nanotubide intermediates, we used the respective carbon nanotubide salt obtained by melting stoichiometric amounts of potassium with HiPco SWCNTs. The bronze-colored salt (KC_4_) was dispersed in absolute DMAc, yielding neatly dispersed carbon nanotubides. It has to be mentioned that strictly H_2_O- and O_2_-free solvents are mandatory for the successful reductive exfoliation and in order to suppress side reactions during the functionalization.^23^ The electrophilic trapping/functionalization sequence was carried out by the addition of varying amounts of benzenediazonium tetrafluoroborate in the potassium : diazonium ratios of 1 : 4, 1 : 1, and 5 : 1. After the addition of the diazonium compound the sample was worked up under aqueous conditions and characterized in detail by means of Raman spectroscopy and thermogravimetric analysis coupled to gas chromatography and mass spectrometry (TG-MS and TG-GC-MS).

It can be expected that based on a retro-functionalization mechanism,^51^ the highest excess of electrophilic trapping species should lead to the highest degree of functionalization and therefore an underlying electronic type selectivity should be easily detectable in the respective SWCNT–Ph_(1:1)_(KC_4_) derivatives, where a 4-fold excess of diazonium cations in relation to applied negative charges was used to quench the carbon nanotubides.

In carbon allotrope characterization, the intensity ratio of the D-mode in relation to the G-mode is commonly used as a measure for the amount of covalently bound addends in the functionalized samples, whereas the radial breathing modes (RBM) are characteristic features for the individual SWCNT chiral indices present in the mixture. Their position in the spectrum only depends on the diameter and on the applied laser excitation energy.^52^–^54^

**Fig. 4:**
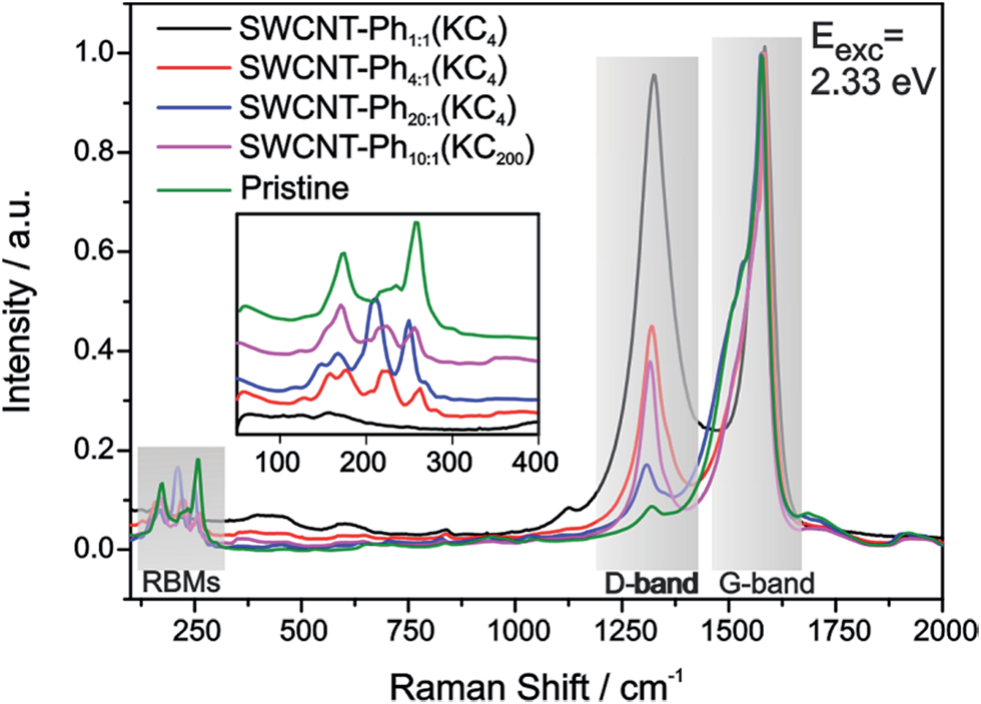
Raman spectra of the reductively arylated SWCNT derivatives (K : C charging ratio 1 : 4 and 1 : 200) with varying trapping electrophile concentration – SWCNT–Ph_C:Ph_(KC_*x*_). Pristine nanotubes shown in green as reference. All spectra are normalized to the maximum amplitude of the G-mode. Inset: RBM region with vertical offset of the spectra for clarity. Laser excitation energy: *E*_exc_ = 2.33 eV.

The mean Raman spectra of the arylated SWCNT derivatives, probed with a laser excitation energy of 2.33 eV, are plotted in Fig. 4 – the spectral information obtained with a laser excitation energy of 1.58 eV is presented in Fig. S4;† spectral data for 14 further excitation energies in the range between 1.59 eV–2.71 eV are presented in the ESI (Fig. S5–S8: RBM region, Fig. S9–S11: D-/G-band region).† Based on the bulk analysis of the obtained *I*_D_/*I*_G_ intensity ratios (based on statistical mean of peak amplitude quotients, *s*. Table S1†) it becomes evident that, as expected, the highest degree of functionalization (*I*_D_/*I*_G_ = 0.97) is obtained for SWCNT–Ph_1:1_(KC_4_) with the highest diazonium excess in relation to applied negative charges (4 : 1), whereas for SWCNT–Ph_20:1_(KC_4_) with a diazonium to charge ratio of 1 : 5 the lowest degree of functionalization with an *I*_D_/*I*_G_ ratio of 0.17 is obtained.

An additional indication for successful addition and a viable feature to probe preferential functionalization of certain nanotubes to a particular tube type is the decrease of RBM intensity. The high concentration of covalently bound addends in SWCNT–Ph_1:1_(KC_4_) leads to an almost complete depletion of the characteristic RBM signature (inset Fig. 4) and, therefore, we focused our electronic type selectivity investigation of the reductive diazotation reaction on sample SWCNT_20:1_(KC_4_) where the RBM fine structure of the spectrum is preserved after the covalent addend binding. Analysis of the Raman resonance profiles allows for the determination of the nanotube indices (*n*,*m*) within the ensemble.^39^,^41^,^55^ This in turn gives the opportunity to choose distinct laser excitation energies addressing either exclusively metallic nanotube families, or exclusively semiconducting ones, or both of them. Thereby, we have a tool to selectively analyze the influence of a functionalization on certain families of carbon nanotubes.

**Fig. 5:**
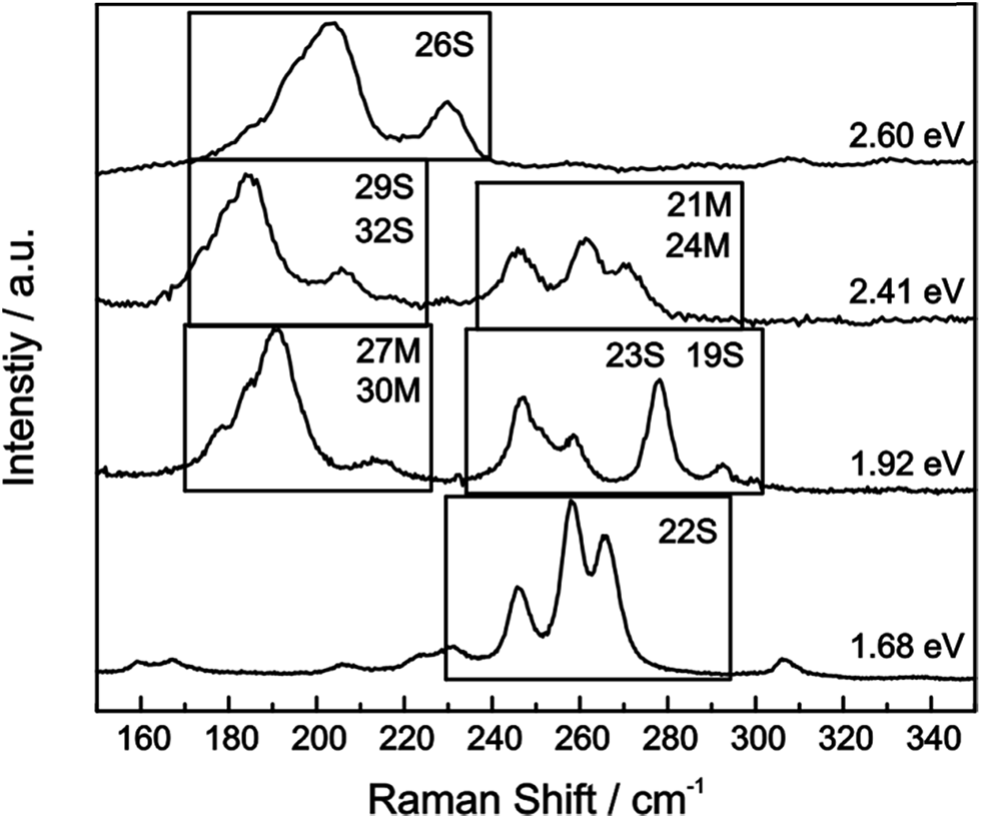
Raman spectra of the radial breathing modes (RBM) of the pristine HiPco sample are plotted for several laser excitation energies. SWCNT (2*n* + *m* = constant) are denoted that have been determined *via* pattern recognition.^39^

For our HiPco SWCNT batch we have predominantly found carbon nanotubes with diameters of around 10 Å. Hence, we address semiconducting tubes with excitation energies between 2.73 and 2.55 eV belonging to the families (2*n* + *m* = constant) 23S and 26S. Between 2.55 eV and 2.30 eV we probe both semiconducting 29S and 32S as well as metallic 21M and 24M carbon nanotubes, whereas around 2.2 eV only metallic 24M and 27M carbon nanotubes are probed. Lower excitation energies will again bring semiconducting tubes in resonance exhibiting larger diameters compared to the metallic tubes in resonance. At laser excitation energies around 1.9 eV, 27M and 30M metallic as well as 23S semiconducting tubes can be excited. From *ca.* 1.75 eV and below only semiconducting tubes are probed. A corresponding family assignment for selected excitation energies can be seen in Fig. 5.

**Fig. 6:**
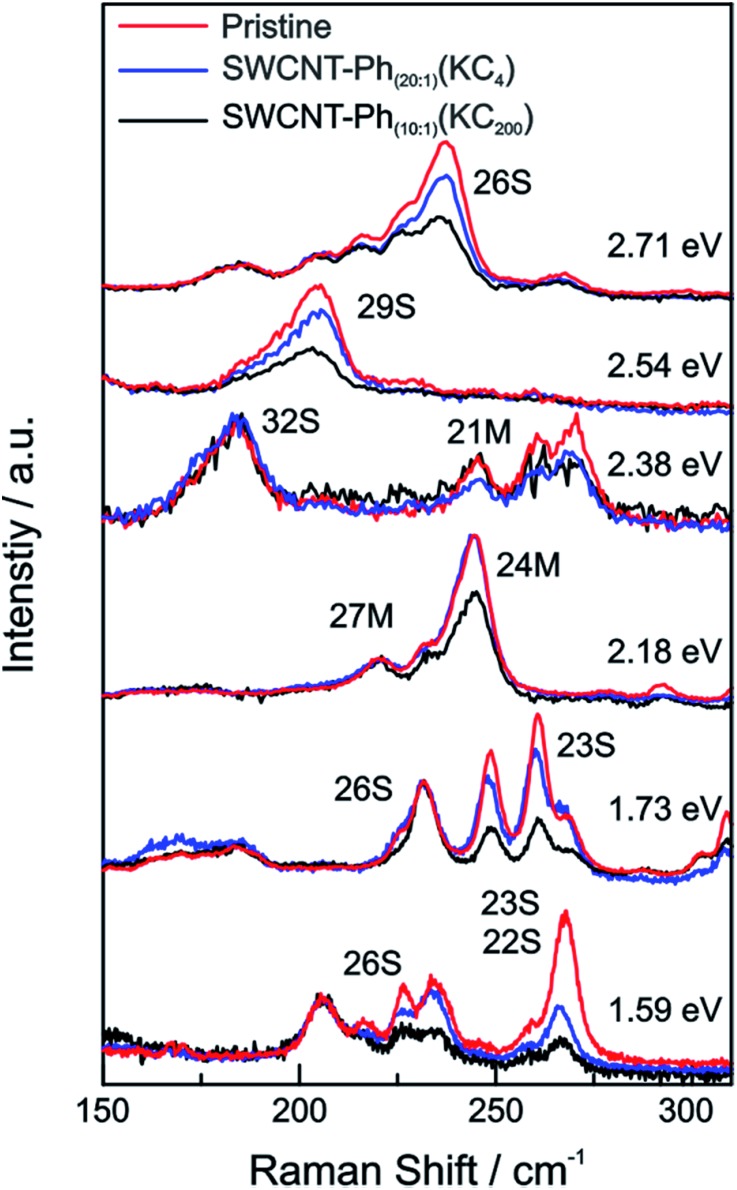
Raman spectra of the RBM region of the pristine starting material (red), SWCNT–Ph_20:1_(KC_4_) (blue), and SWCNT–Ph_10:1_(KC_200_) (black) after reductive diazotation plotted for various laser excitation energies (given next to the spectra). For each excitation energy the Raman intensities are normalized to the RBM with the lowest frequency, corresponding to the nanotube with the largest diameter.

**Fig. 7:**
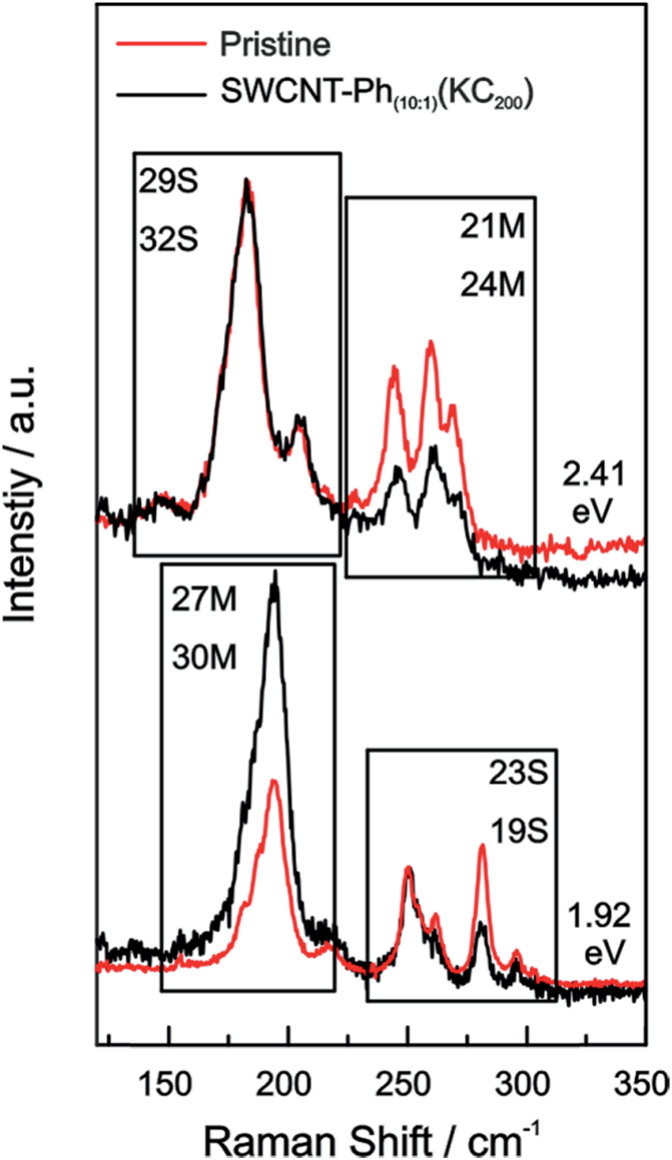
Raman spectra of the RBMs of the pristine starting material (red) and SWCNT–Ph_10:1_(KC_200_) (black) after reductive diazotation plotted for two excitation energies (top: 2.41 eV; bottom: 1.92 eV). In both cases semiconducting tubes are in resonance alongside metallic ones. The spectra are normalized to the semiconducting carbon nanotube with the lowest RBM Raman shift (largest diameter).

**Fig. 8:**
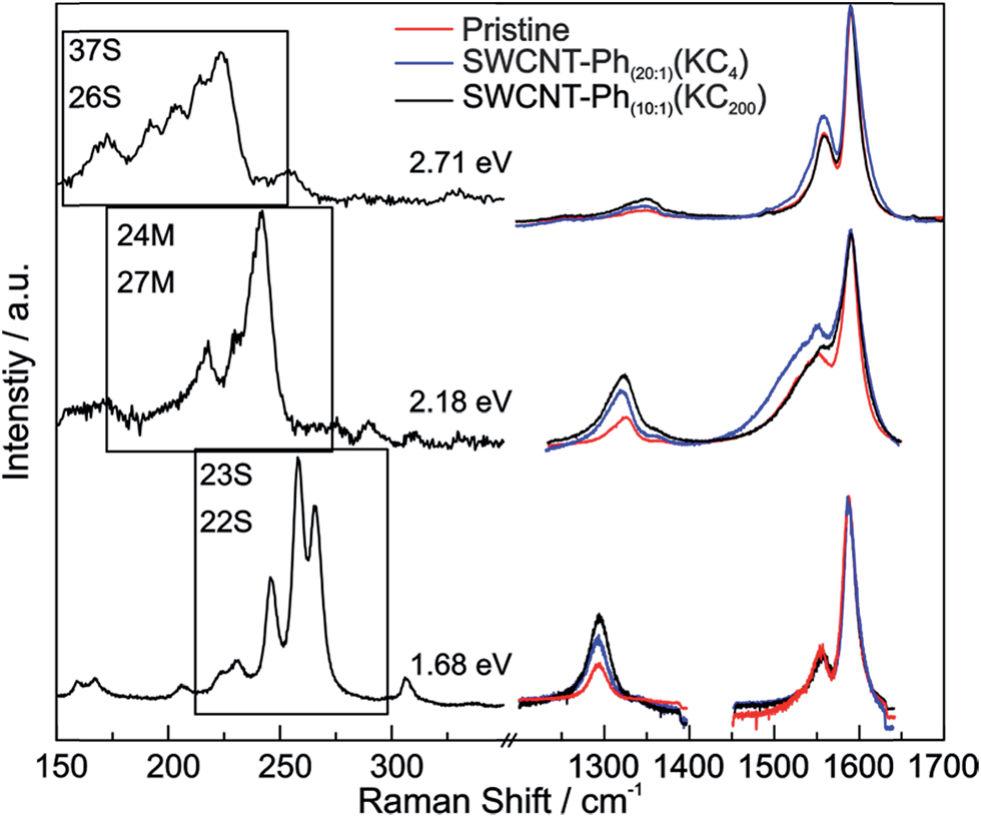
Left: Raman spectra of the RBMs for excitation wavelengths probing only semiconducting (37S and 26S at 2.71 eV, 23S and 22S at 1.68 eV) or metallic SWCNTs (24M and 27M at 2.18 eV). Right: corresponding Raman spectra of the G and D modes. Pristine starting material (red), SWCNT–Ph_20:1_(KC_4_) (blue), and SWCNT–Ph_10:1_(KC_200_) (black) after reductive diazotation. The D- and G-band regions of the spectra are normalized to the amplitude of the G^+^ mode.

From the Raman analysis of SWCNT–Ph_*n*:1_(KC_*x*_) we find several statements: (i) after functionalization, the RBM intensities drop notably. Further, our experimental data indicates that a covalent functionalization does not affect the optical transition energies significantly, *i.e.*, for a constant excitation energy we probe the same nanotube types before and after the functionalization. (ii) Carbon nanotubes with smaller diameters are generally more sensitive towards a functionalization compared to those with larger diameters. This can be identified by the significantly stronger depletion of smaller nanotubes' RBMs (higher Raman shifts) in the corresponding Raman spectra (Fig. 6), where the RBM spectra are normalized in intensity to the mode of the nanotube with the largest diameter (smallest Raman shift). This well-known behavior applies for both metallic and semiconducting carbon nanotubes and can be explained by the increased ring strain. (iii) In Fig. 7 Raman spectra are depicted in which both metallic and semiconducting tubes are in resonance. The RBM intensities in the upper part indicate that metallic carbon nanotubes are affected by the functionalization to a larger extent compared to semiconducting tubes. In contrast, the spectra in the lower part (Fig. 7) indicate the opposite behavior, *i.e.*, semiconducting tubes seem to be affected to a larger extent by the functionalization. However, a direct comparison is unfeasible since the effect of a metallicity-selective functionalization is superimposed by the dominant influence of diameter-selective functionalization. To circumvent this aspect, we compare the D-mode intensities where exclusively metallic (2.18 eV) or semiconducting (2.71 eV, 1.68 eV) tubes are in resonance with the laser excitation energy *E*_exc_ (see Fig. 8). For *E*_exc_ = 2.71 eV (1.68 eV) the tube diameters are larger (smaller) compared to the ones probed for *E*_exc_ = 2.18 eV. In all cases we observe an increase of the D-mode intensity after functionalization. However, the intensity increase for metallic carbon nanotubes is larger compared to that of semiconducting tubes with larger diameters and almost equal to smaller semiconducting carbon nanotubes. This direct comparison indicates that the functionalization rather affects metallic carbon nanotubes than semiconducting ones.

**Fig. 9:**
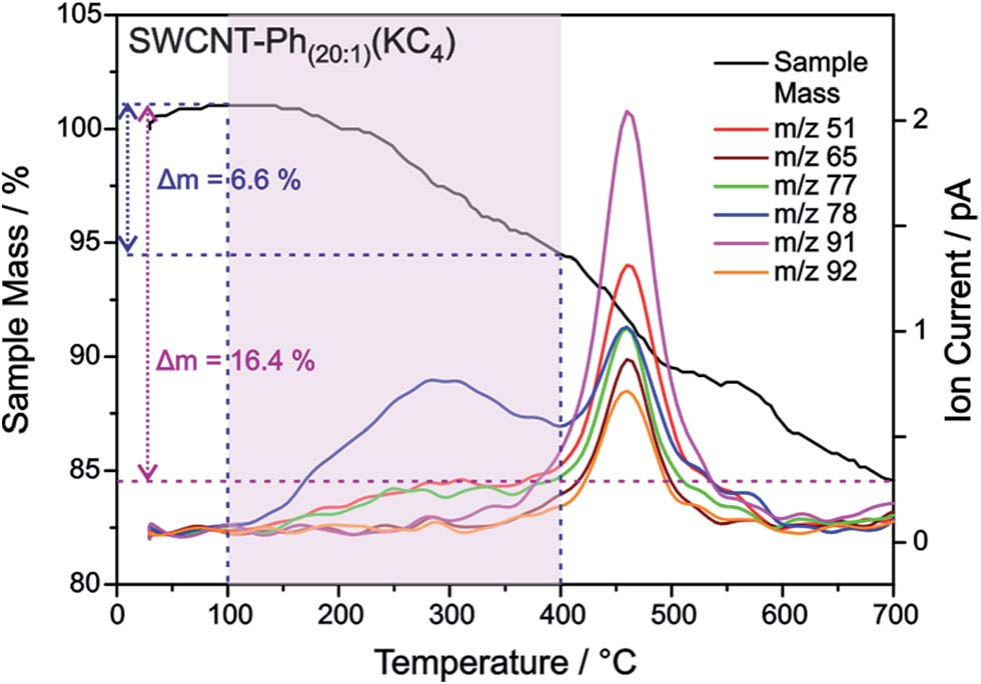
TGA mass loss and ion current traces for reductively arylated sample SWCNT–Ph_20:1_(KC_4_) – *m*/*z* 78 can be attributed to the protonated detached aryl moieties C_6_H_6_˙^+^ (other fragmentation products are provided in Fig. S13†).

**Scheme 2:**
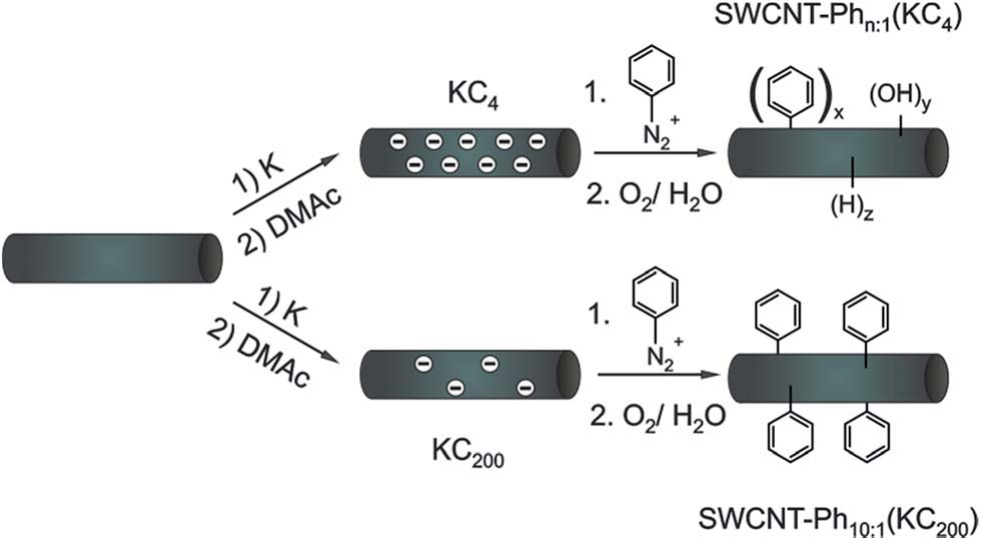
Reaction scheme for two different charging scenarios. For SWCNT–Ph_20:1_(KC_4_), the applied negative charges are not fully consumed by the addition of the trapping electrophile. In the course of the subsequent aqueous work-up hydrogen and oxygen functionalities are introduced due to the residual charges. In the case of SWCNT–Ph_10:1_(KC_200_) benzenediazonium tetrafluoroborate is used in excess. This leads to complete charge neutralization before work-up.

As suggested by Laudenbach *et al.*,^56^*I*_D_/*I*_G_ values can be utilized to compare the degree of functionalization for different excitation energies. In order to quickly compare the reaction success and to facilitate the determination of nanotube type-dependent selectivity, it is useful to introduce a new term Δ*D*/G^±^ defined by eqn (2):2
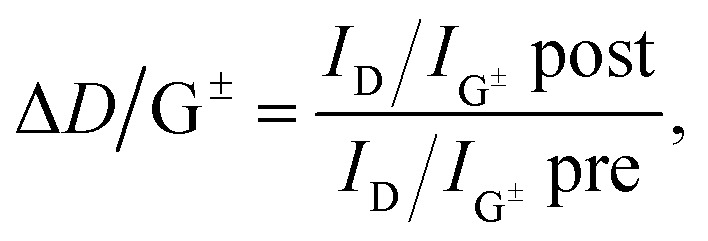
with *I*_D_/*I*_G^±^_post being the *I*_D_/*I*_G_ ratio after functionalization and *I*_D_/*I*_G^±^_pre being the *I*_D_/*I*_G_ ratio before functionalization (*i.e.* of pristine nanotubes). G^±^ refers to the fact that the G^+^ (G^–^) mode needs to be taken into account when regarding the reactions success of semiconducting (metallic) SWCNTs. Corresponding values are listed in Table 4. The highest Δ*D*/G^±^ can be found for the metallic 24M and 27M carbon nanotubes excited with 2.18 eV for both samples, while the value is uniformly lower for semiconducting nanotubes (2.71 and 1.68 eV). The very low value for SWCNT–Ph_20:1_(KC_4_) is due to the fact that the line width of the G^+^ mode increases significantly after the functionalization. These results show a preferred functionalization of metallic SWCNTs and are in good agreement with the predictions of our DFT calculations. However, we wanted to also obtain a clearer picture of the actual chemical nature of the reductively bound addend in our sample. Raman spectroscopy, although potentially providing direct insight into the introduction of sp^3^-hybridized carbon lattice atoms due to the covalent addend binding, does not yield any information about the chemical nature of the bond entities. For this purpose, the reductively arylated derivatives have been analyzed by thermogravimetric analysis coupled to mass spectrometry (TG-MS). Here, the sample is heated under an inert gas atmosphere and the thermal detachment of the covalently bound aryl moieties can be constantly monitored by mass spectrometry. The obtained mass loss of the sample can then be used for the calculation of the degree of functionalization.^5^,^24^ SWCNT–Ph_20:1_(KC_4_) exhibits a total mass loss of 16.4% (Fig. 9) – for comparison the respective TG-MS data obtained for the other SWCNT derivatives are presented in Fig. S12† and Table S1† – and according to the recorded MS traces, two distinct temperature regimes for the detection of aryl fragments can be identified. As has recently been demonstrated for arylated graphene derivatives,^57^ the detected masses (for a respective assignment of the detected masses see Fig. S13†) around 450 °C can be attributed to small aromatic fragments derived from the decomposition of the carbon framework structure. The detachment of the sidewall-bound phenyl ring (C_6_H_6_˙^+^: mass-to-charge-ratio (*m*/*z*) = 78) takes place at around 100–400 °C (note: cleaved phenyl radicals abstract hydrogen from their environment and are detected as intact benzene rings in both TG-MS and TG-GC-MS.^57^). With the knowledge of the exact cleavage temperature of the covalent addends at hand, a degree of functionalization (DoF) can be approximated for all samples (details for the calculation see Chapter S6 and formula (S1) in the ESI†). For the samples synthesized from KC_4_, the DoF is strongly influenced by the amount of used diazonium salt (see Table S1†). While SWCNT–Ph_(1:1)_(KC_4_) reached a relatively high grafting ratio of 3.8%, SWCNT–Ph_(1:4)_(KC_4_) reached 2.1% only, and SWCNT–Ph_(20:1)_(KC_4_) merely 1.2%. The first two products seem to be cleanly phenyl-functionalized, whereas the TG-MS results of the latter sample (Fig. 9) indicate that the remaining negative charges, due to a sub-stoichiometric amount of the diazonium trapping reagent, lead to a side functionalization in the course of the subsequent aqueous work-up, where oxygen and hydrogen functionalities are created on the SWCNT sidewalls in addition to the phenyl moiety (Scheme 2).^23^ Thus, the corresponding DoF of SWCNT–Ph_(20:1)_(KC_4_) should be treated with care. Nevertheless, all obtained DoFs are in good agreement with the Raman results, in which the high *I*_D_/*I*_G_ ratio of SWCNT–Ph_(1:1)_(KC_4_) indicates strong functionalization, decreasing with lower amounts of diazonium reagent. Most interestingly, mentioned side product formation does not superimpose any initial electronic type selectivity of the functionalization and the results obtained by Raman analysis are a strong indication that the side product formation likewise proceeds in an electronic type selective fashion.

**Table 4:** Δ*D*/G^±^ values for selected laser excitation energies. The analysis has been carried out according to Laudenbach *et al.*^56^ The data corresponds to the spectra depicted in Fig. 8. (sc) and (m) indicate that exclusively semiconducting and metallic nanotubes, respectively, are in resonance

	*E*_exc_ = 2.71 eV (sc)	*E*_exc_ = 2.18 eV (m)	*E*_exc_ = 1.68 eV (sc)
SWCNT–Ph_(20:1)_(KC_4_)	Δ*D*/G^+^: 1.05, Δ*D*/G^–^: 1.44	Δ*D*/G^+^: 0.16, Δ*D*/G^–^: 1.86	Δ*D*/G^+^: 1.50, Δ*D*/G^–^: 1.70
SWCNT–Ph_(10:1)_(KC_200_)	Δ*D*/G^+^: 1.44, Δ*D*/G^–^: 2.37	Δ*D*/G^+^: 2.46, Δ*D*/G^–^: 2.77	Δ*D*/G^+^: 1.05, Δ*D*/G^–^: 1.44

**Fig. 10:**
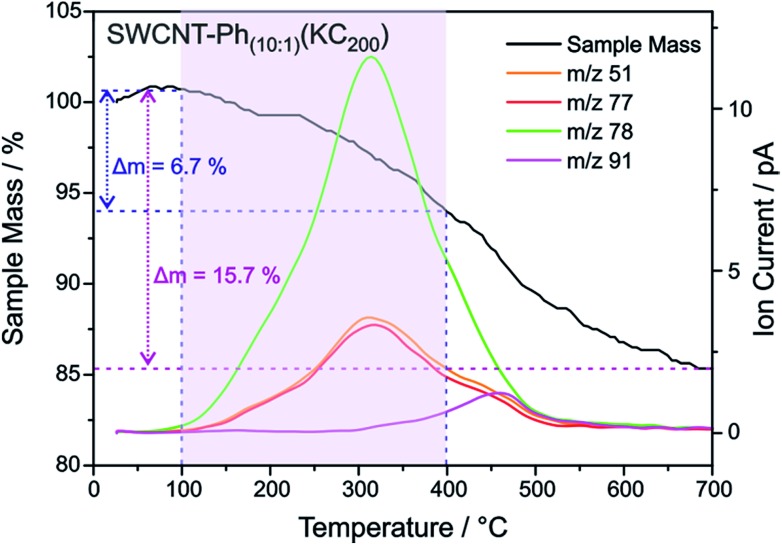
TGA-mass loss and ion current traces for reductively arylated sample SWCNT–Ph_10:1_(KC_200_).

As outlined above, we have recently found that low potassium charging concentrations (K : C < 1 : 200) lead to a selective charging of metallic SWCNT helicities.^29^ Therefore, we wanted to investigate whether this could be utilized in a cooperative manner to increase the amount of selectively functionalized SWCNTs and whether a side product formation could be suppressed by the use of an equimolar amount of trapping reagent to consume the applied negative charges in full extent. Our selection of the reductive conditions is furthermore driven by the results obtained by the DFT calculations, where a supercell consists of several hundred carbon atoms and one single negative charge (see Table 1). The binding energies (*E*_b_) for the diazonium cation with the negatively charged SWCNT species are on average higher for metallic tubes, the maximum differences being about 0.3–0.5 eV. This is the same energy window that has been calculated for the difference of the adsorption energies of potassium atoms on negatively charged metallic SWCNTs in relation to semiconducting species.^29^ This selective charging of metallic SWCNT species is expected to take place up to a potassium to carbon ratio of 1 : 200. With this type of charged starting material we should in principle be able to exploit two cumulative selectivity-determining parameters – (a) selective charging of metallic SWCNT helicities and (b) preferred energetic interaction of the diazonium cation with negatively charged metallic SWCNT species. The Raman screening (Fig. 4) of the respective sample (SWCNT–Ph_10:1_(KC_200_)) clearly indicates that a reductive arylation under these low charging conditions is possible – obtained *I*_D_/*I*_G_ ratio: 0.39 for *E*_exc_ = 2.33 eV (further Raman information is presented in the ESI†). Moreover, the characteristic RBM signature of the carbon nanotubes is also preserved under these conditions, rendering the sample accessible for an in-depth Raman investigation – see below. Regarding the TG-MS characterization data, SWCNT–Ph_(10:1)_(KC_200_) exhibits a total mass loss of around 15.7% (Fig. 10). Here, the characteristic masses of the bound benzene moiety (*m*/*z* 78, C_6_H_6_^.+^; *m*/*z* 77, C_6_H_5_^+^; *m*/*z* 51, C_4_H_3_^+^) can be detected as main peaks at around 310 °C, which, as outlined above, is the typical temperature regime for the cleaving of covalently attached aryl addends. In addition, SWCNT–Ph_(10:1)_(KC_200_) shows only minor framework degradation as opposed to the reductively arylated sample with a sub-equimolar concentration of the diazonium cation SWCNT–Ph_20:1_(KC_4_). Therefore, it can be concluded that under the chosen conditions the reductive arylation of the carbon nanotubide intermediates proceeds in a clean fashion, hinting that the calculated DoF of 1.2% is a good approximation, unlike for sample SWCNT–Ph_(20:1)_(KC_4_). This is also consistent with a higher *I*_D_/*I*_G_ ratio in the Raman spectra (Fig. 4).

**Fig. 11:**
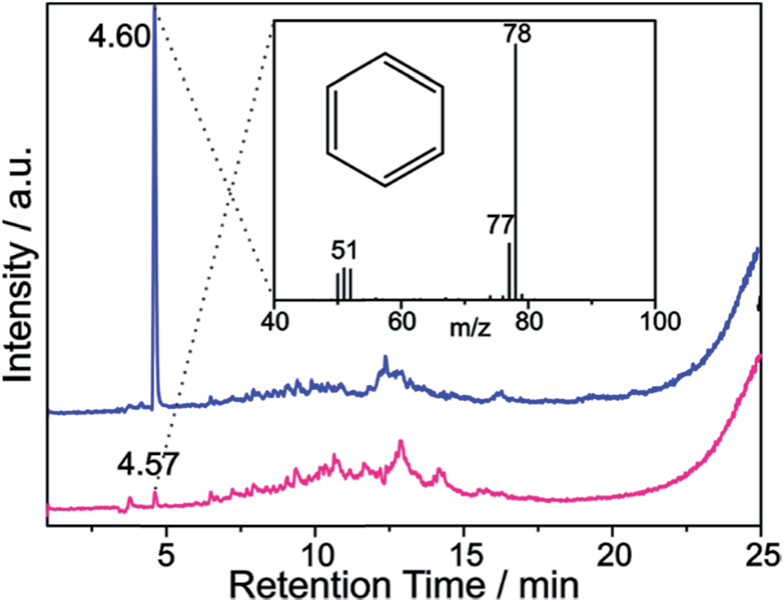
GC elugram of SWCNT–Ph_(10:1)_(KC_200_) (blue trace) and SWCNT–Ph_(20:1)_(KC_4_) (red trace) – vertical offset for clarity. GC injection temperature: 280 °C. Inset: MS spectrum of the peak at 4.6 min retention time, indicative for the thermally detached phenyl moieties bound to the SWCNT sidewalls.

These findings are further corroborated by a gas chromatographic separation and characterization of the thermally detached gaseous components. In Fig. 11 the respective GC elugrams of SWCNT–Ph_10:1_(KC_200_) and SWCNT–Ph_20:1_(KC_4_) are compared. For SWCNT–Ph_(10:1)_(KC_200_) the elugram clearly shows benzene as only analyte peak at an injection temperature of 280 °C. Biphenyl or longer polyphenyl chains obtained by a radical recombination of the intermediately generated aryl radicals were not detected. For SWCNT–Ph_(20:1)_(KC_4_), only a minor fraction of detached benzene can be found at an injection temperature of 280 °C (Fig. 11 – red trace). Furthermore, at an injection temperature of 480 °C, several aromatic components like toluene, indicative for a SWCNT framework decomposition,^57^,^58^ can be detected for SWCNT–Ph_(20:1)_(KC_4_) (Fig. S14†). These results clearly underline that SWCNT–Ph_(10:1)_(KC_200_) represents an appropriate sidewall-functionalized SWCNT sample and is suitable for analysis of the underlying electronic type selectivity by means of resonance profile analysis of the Raman RBM region. Fig. 8 and Table 4 enable to directly compare the *I*_D_/*I*_G_ ratios of the two systems SWCNT–Ph_20:1_(KC_4_) and SWCNT–Ph_10:1_(KC_200_). It can be seen that the *I*_D_/*I*_G_ ratios for SWCNT–Ph_10:1_(KC_200_) are larger for all laser excitation energies, probed both for metallic and semiconducting carbon nanotubes, indicating that the SWCNT–Ph_10:1_(KC_200_) functionalization is more efficient compared to the SWCNT–Ph_20:1_(KC_4_). This is in accordance with TG-MS results and nicely confirms our rationale that the two electronic type determining parameters can be used in a cooperative fashion.

## Conclusions

The reaction of neutral SWCNTs with diazonium salts proceeds with a high selectivity towards metallic carbon nanotube species, like previously observed in Birch type charged SWCNT samples. In our present joint theoretical and experimental study, we were able to elucidate the underlying selectivity of this reductive diazotation reaction. Based on our DFT calculation results, a preferred interaction of the diazonium cation with charged, metallic carbon nanotubes could be identified as one major component in the electronic type selective functionalization of reductively charged metallic SWCNT helicities. Our present experimental results on the reductive arylation of carbon nanotubes obtained by the solid state intercalation approach with potassium in varying concentrations nicely confirm the predicted preferred functionalization of metallic carbon nanotubes. The Raman-based analysis of the electronic type selectivity is complicated by the fact that small SWCNTs in general exhibit a higher reactivity towards covalent addition reactions. By using different excitation energies with several combinations of nanotubes with different electronic types and diameters in resonance we were able to deconvolve these effects and succeeded in determining the different carbon nanotube helicities within the whole ensemble as well as proving the preferential functionalization of the metallic SWCNT helicities. Moreover, we could also show by a detailed thermogravimetric analysis of the reaction products that if sub-stoichiometric amounts of trapping reagents are used, the remaining negative charges on the SWCNTs lead to a side product formation introducing oxygen and hydrogen functionalities. This side product formation does not superimpose the initial selectivity for metallic SWCNTs. When low potassium charging concentrations are used (K : C < 1 : 200), the amount of arylated metallic carbon nanotubes can be increased by cooperatively utilizing the selective charging of metallic species prior to the reaction with diazonium cations.

On the basis of our results the underlying parameters for a selective functionalization of metallic SWCNTs with diazonium cations could be elucidated. In ongoing studies, the DFT approach can now be extended to further trapping electrophiles, like alkyl and aryl halides and λ^3^-iodanes.

## Conflicts of interest

The authors declare that there are no conflicts of interest.

## Supplementary Material

Supplementary information
