# Supplementary material for: Reductive diazotation of carbon nanotubes: an experimental and theoretical selectivity study
Source: Chem Sci. 2018 Oct 22;10(3):706–17. doi: 10.1039/c8sc03737j (PMC6340405; doi:10.1039/c8sc03737j)
Supplement: Supplementary file 1 [file SC-010-C8SC03737J-s001.pdf]

Electronic Supplementary Material (ESI) for Chemical Science.
This journal is © The Royal Society of Chemistry 2018

Electronic Supplementary Information (ESI) for

Reductive Diazotation of Carbon Nanotubes: An Experimental and Theoretical Selectivity Study

Milan Schirowski,[†a] Christoph Tyborski,[b] Janina Maultzsch,[b,c] Frank Hauke,[a] Andreas Hirsch[a*] and Jakub Goclon[†*d]

[a]Chair of Organic Chemistry II & Joint Institute of Advanced Materials and Processes, Friedrich-Alexander University of Erlangen-Nürnberg, Nikolaus Fiebiger Straße 10, 91058 Erlangen, Germany.

[b]Institut für Festkörperphysik, Technische Universität Berlin, Hardenbergstraße 36, 10623 Berlin, Germany.

[c]Chair of Experimental Physics, Friedrich-Alexander University of Erlangen-Nürnberg, Staudtstr. 7, 91058 Erlangen, Germany.

[d]Institute of Chemistry, University of Bialystok, Ciolkowskiego Str. 1K, 15-245 Bialystok, Poland.

*E-mail: j.goclon@uwb.edu.pl; andreas.hirsch@fau.de

[†]Both authors contributed equally to the present study.

S1. *Ab initio* Calculations

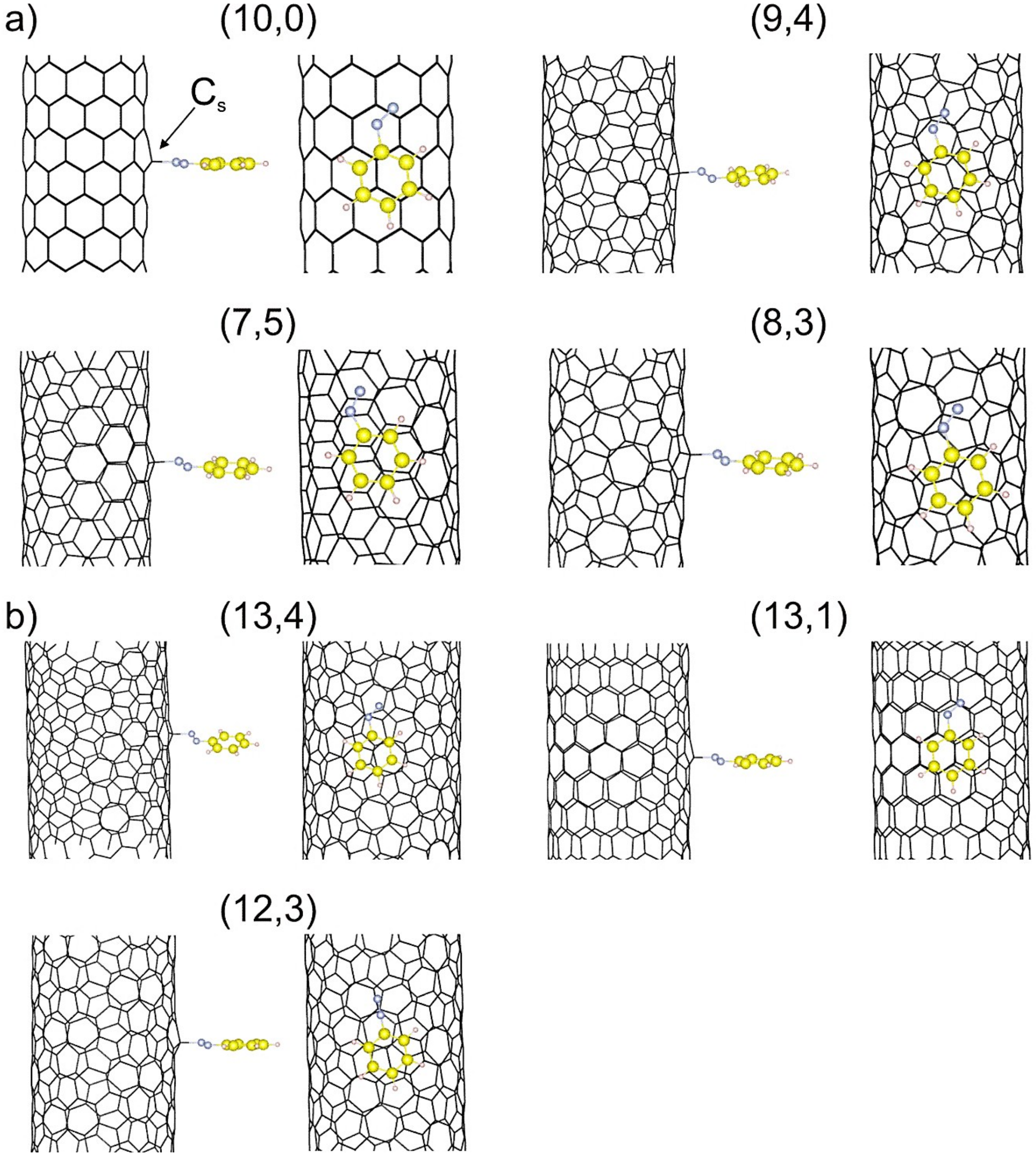

Figure S1: The perpendicular (L) and parallel (II) arrangement of the diazonium aryl cation with the charged semiconducting (a) and metallic (b) SWCNTs after geometry optimization. C_S denotes the carbon atom at the functionalization site. Carbon atoms of the attached cation are yellow, nitrogen atoms are blue and hydrogen atoms are pink.

In our previous work[1] the three symmetric adsorption sites of the parallel orientation of benzene on SWCNT sidewalls were considered: hollow (H), bridge (B) and top (T) using PBE+D2 exchange-correlation functional. According to the calculations, the most stable site is the hollow one. In the present work, initially, the diazonium aryl cation was always put at this site at about 3.5 Å distance to maximize the interactions between the SWCNT and the diazonium group. As a result, the final geometries obtained for the parallel (II) arrangement are the compromise between the hollow (H) position of the aryl ring from PhN_2^+ and the most favorable orientation of the diazonium group.

S2. Electronic Properties of Studied Systems

The band structures of the studied systems are shown in Fig. S2. The occupied band close to/at the Fermi level (spin-up) is non-dispersive. The formation of such a band is related with a strong localization of the wave function (singly occupied molecular orbital (SOMO) of the PhN_2 radical, see Sec. S3).

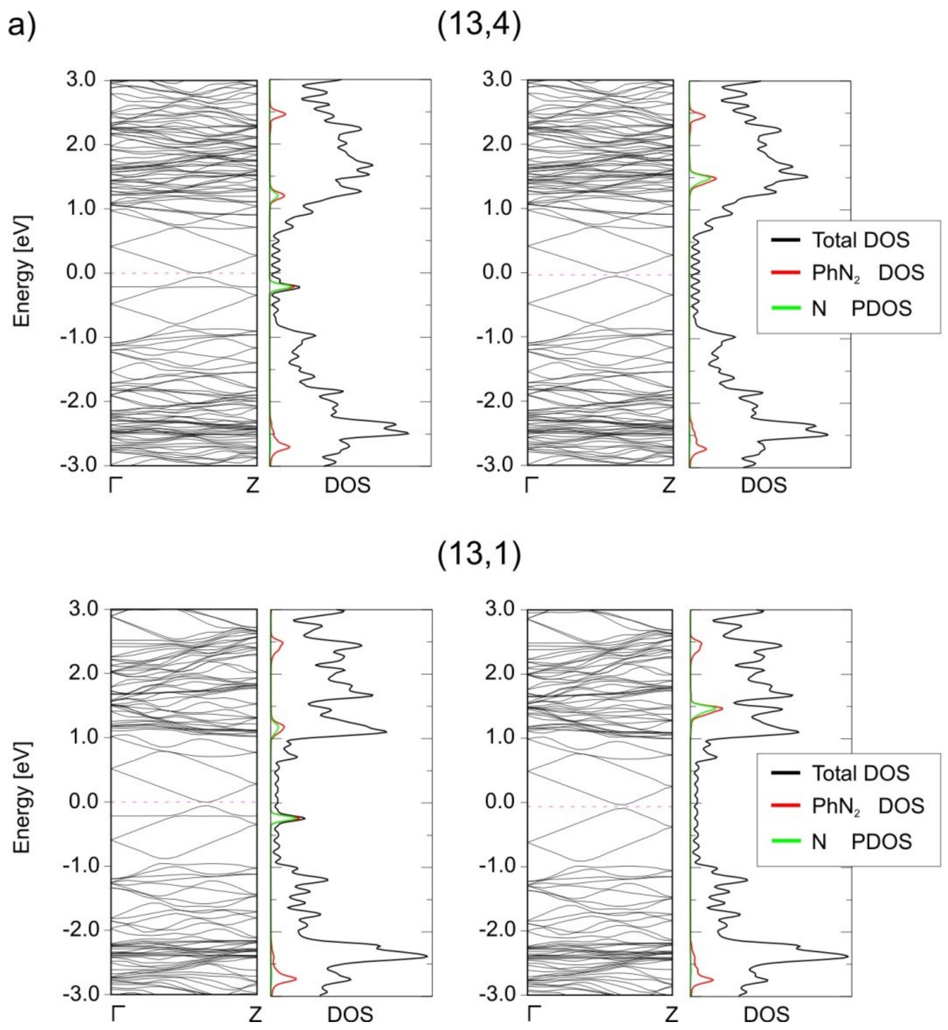

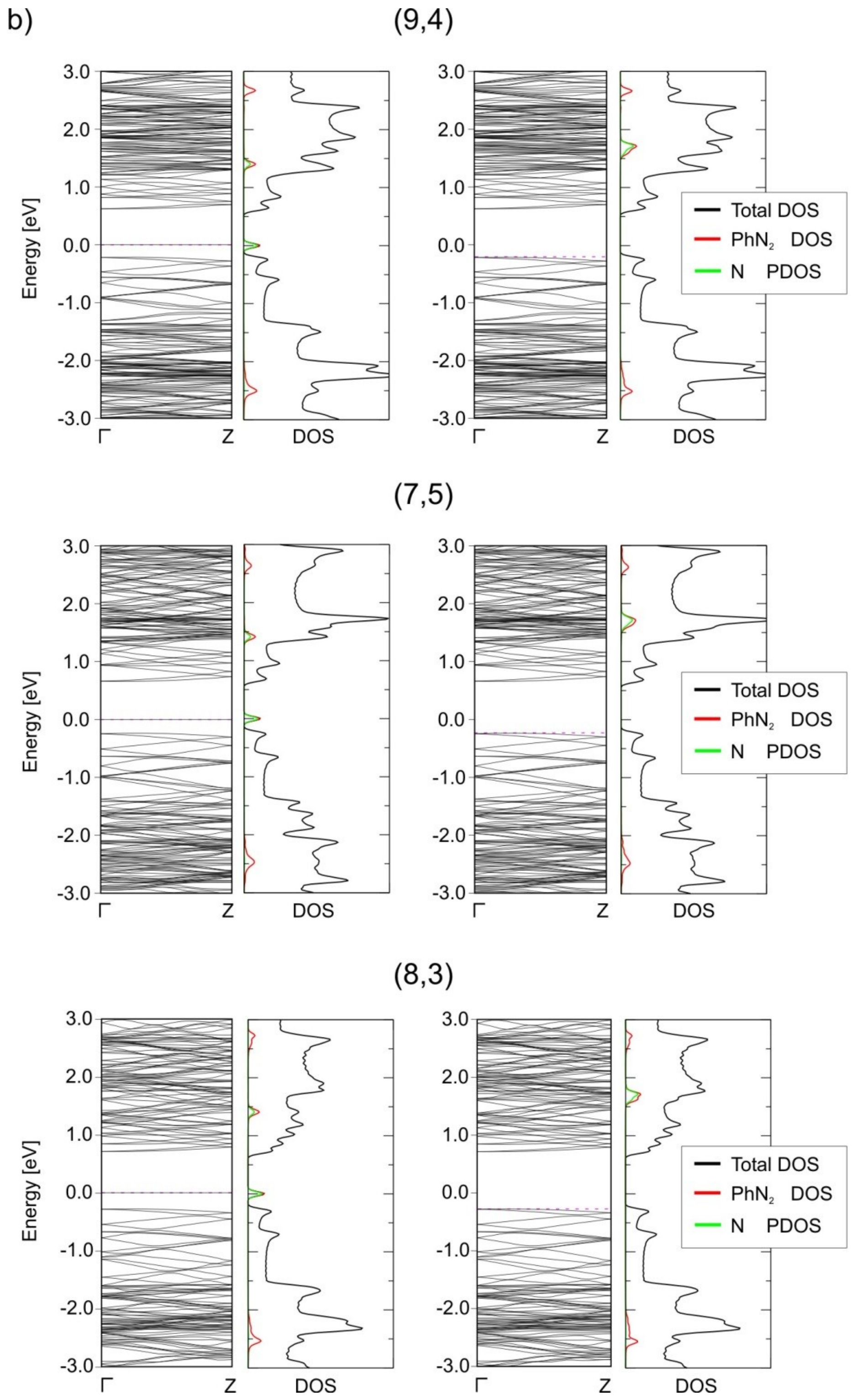

Figure S2: Band structures and density of states (DOS) of the metallic (a) and semiconducting (b) systems with the parallel (II) arrangement. For the spin-polarized systems the left side of the graph corresponds to the spin-up, the right side to the spin-down electronic contribution. The pink dashed lines correspond to the maximally occupied spin-up and spin-down channels. The Fermi energy is set to 0 eV.

S3. Electronic Properties of PhN_2 and PhN_2^+

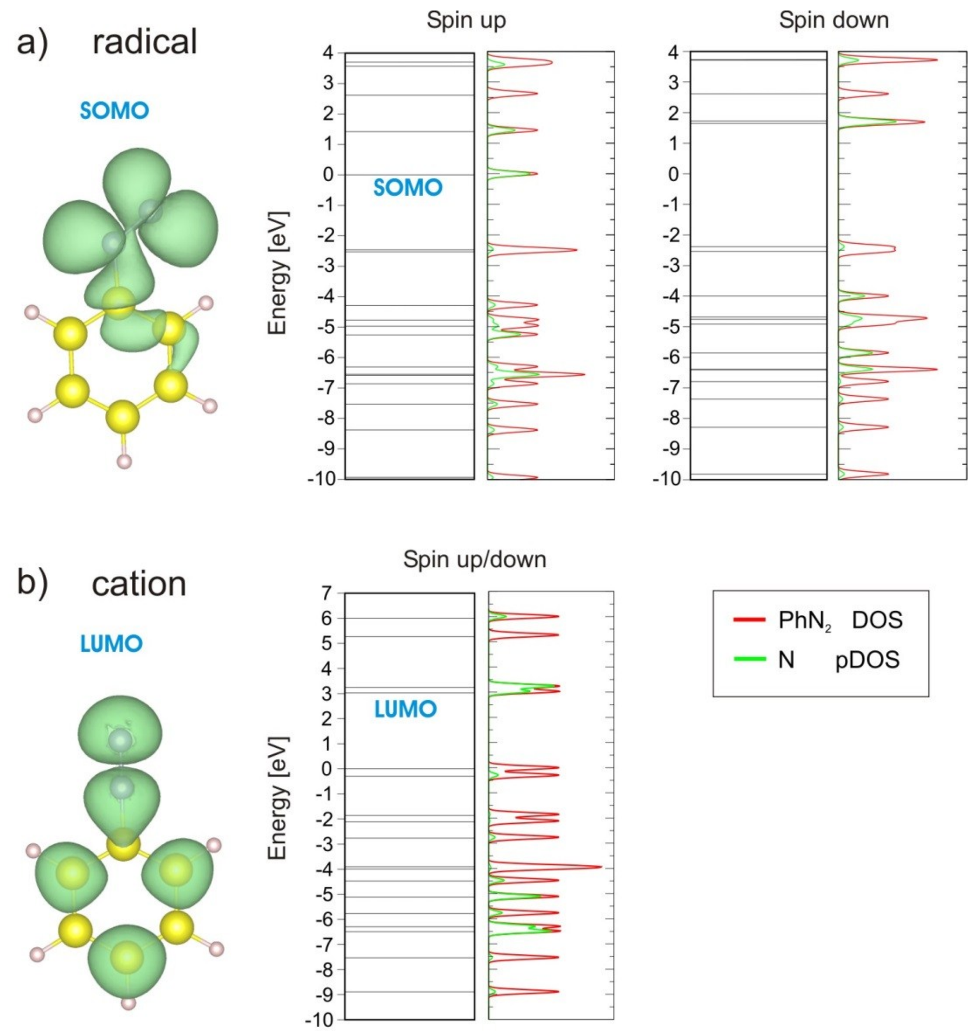

Figure S3: PBE+D2 optimized structures of the $PhN_2^{\cdot}$ radical (a) and PhN_2^+ cation (b) in the gas phase together with the contours of the electron density of the Γ-point wave function of the singly occupied molecular orbital (SOMO) and lowest unoccupied molecular orbital (LUMO), respectively. Molecular levels, total and projected density of states (DOS) are presented for easy comparison with those from the SWCNT-PhN_2 systems. Carbon atoms are depicted in yellow, nitrogen atoms in blue and hydrogen atoms in pink.

S4. Complementary Raman Spectra of SWCNT-$Ph_{(C:Ph)}(KC_x)$

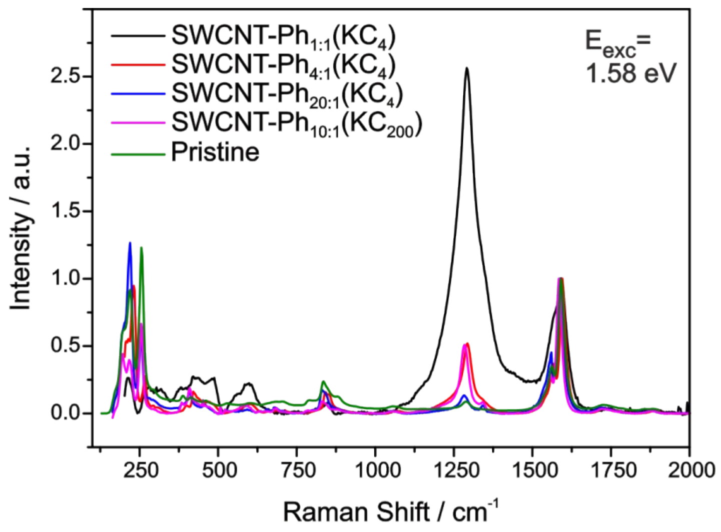

Figure S4: Raman spectra of the reductively arylated SWCNT derivatives (K:C charging ratio 1:4) with varying trapping electrophile concentration – SWCNT-$Ph_{C:Ph}(KC_4)$ in comparison to the low-charged sample (K:C charging ratio 1:200). All spectra normalized to the G-mode intensity. Laser excitation energy: E_{exc} = 1.58 eV.

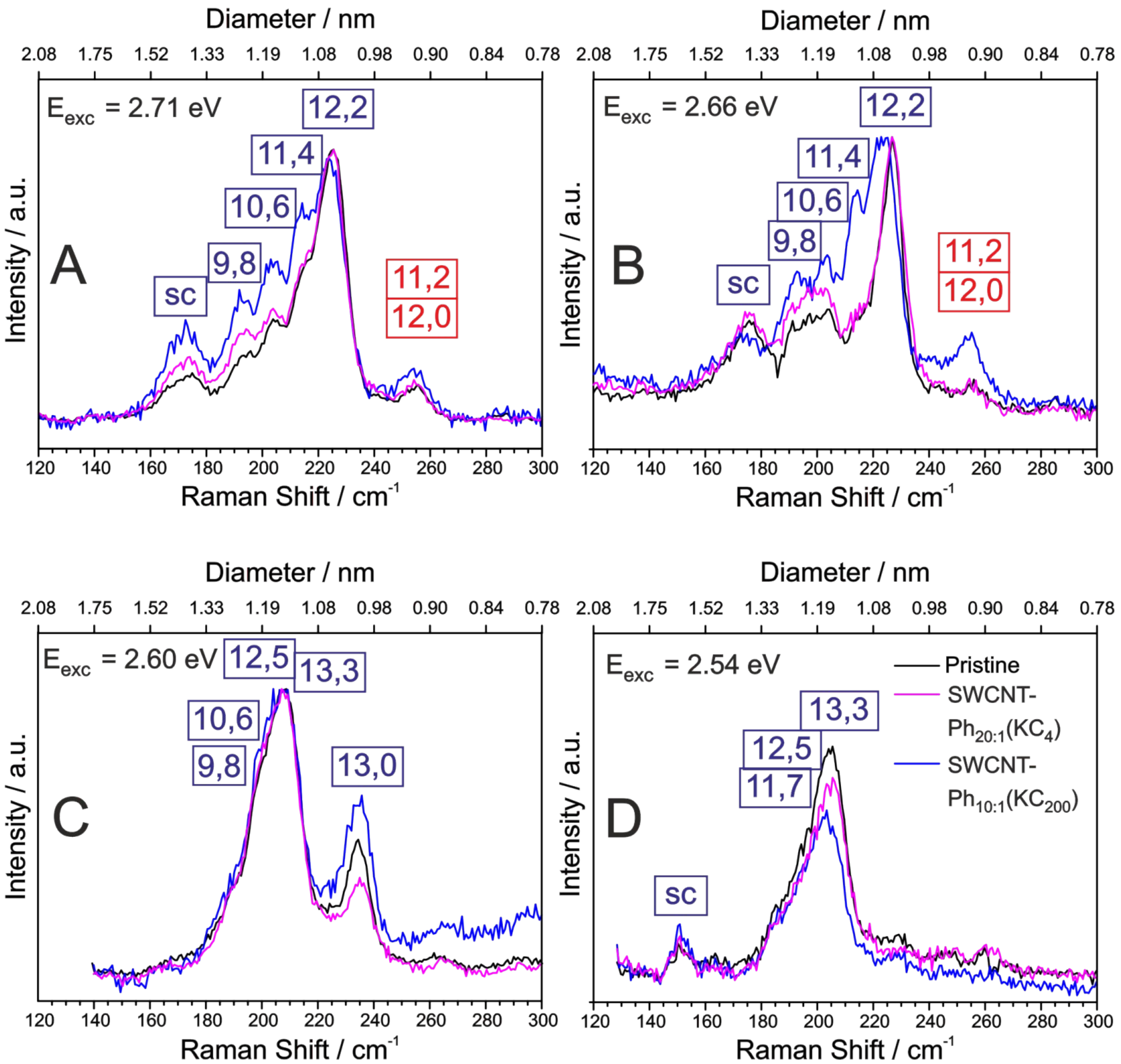

Figure S5: Normalized Raman spectra of the RBM region of pristine HiPco SWCNTs (black) alongside compound SWCNT-$Ph_{20:1}(KC_4)$ (pink) and SWCNT-$Ph_{10:1}(KC_{200})$ (blue) at 2.71 eV (A), 2.66 eV (B), 2.60 eV (C), and 2.54 eV (D) excitation laser energy. Each peak is assigned with nanotube indices (m,n) in red (metallic SWCNTs) or purple (semiconducting SWCNTs), when precise assignment was possible, otherwise with “m” or “sc”, respectively.

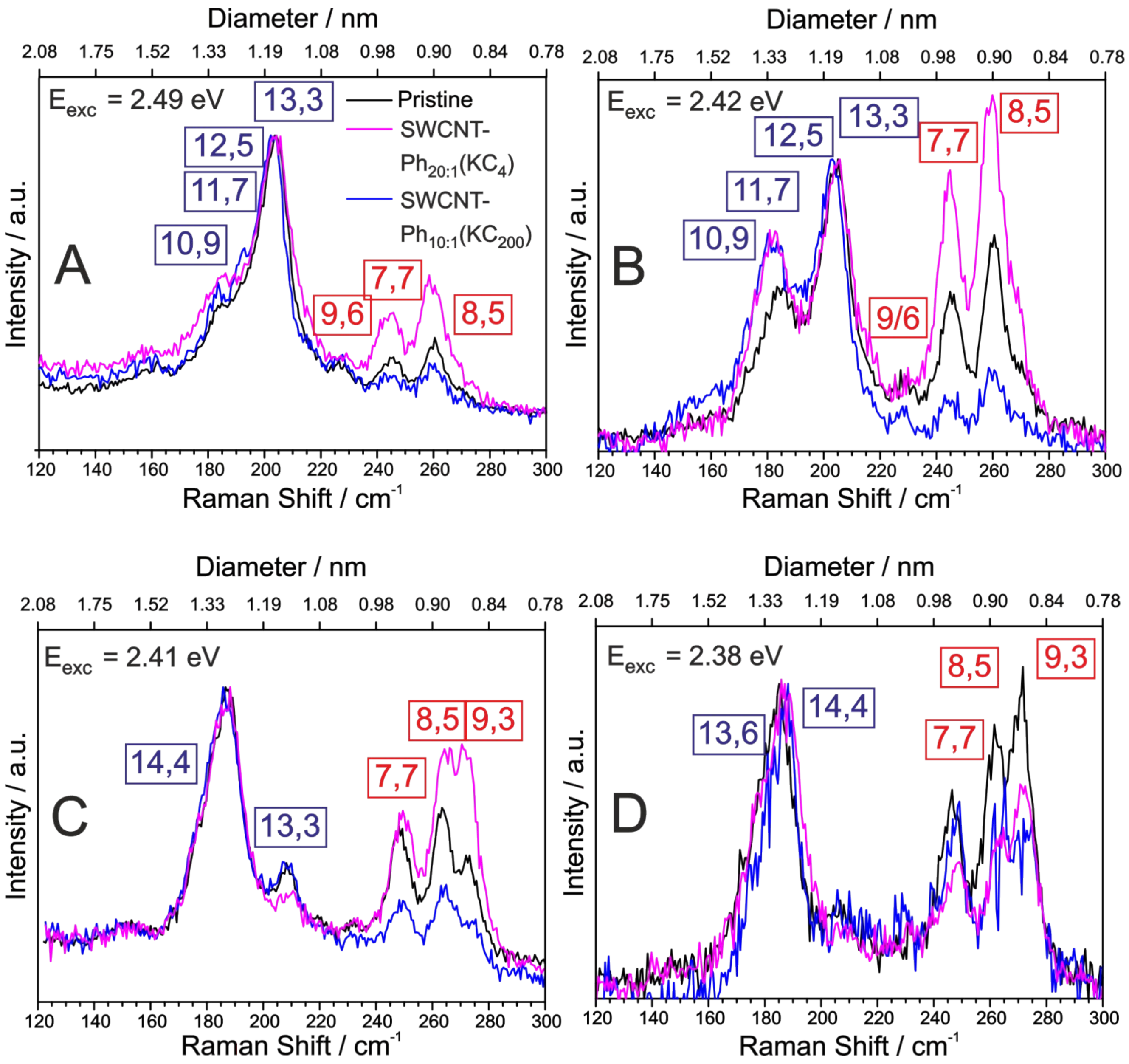

Figure S6: Normalized Raman spectra of the RBM region of pristine HiPco SWCNTs (black) alongside compound SWCNT-Ph$_{20:1}$(KC$_4$) (pink) and SWCNT-Ph$_{10:1}$(KC$_{200}$) (blue) at 2.49 eV (A), 2.42 eV (B), 2.41 eV (C), and 2.38 eV (D) excitation laser energy. Each peak is assigned with nanotube indices (m,n) in red (metallic SWCNTs) or purple (semiconducting SWCNTs), when precise assignment was possible, otherwise with "m" or "sc", respectively.

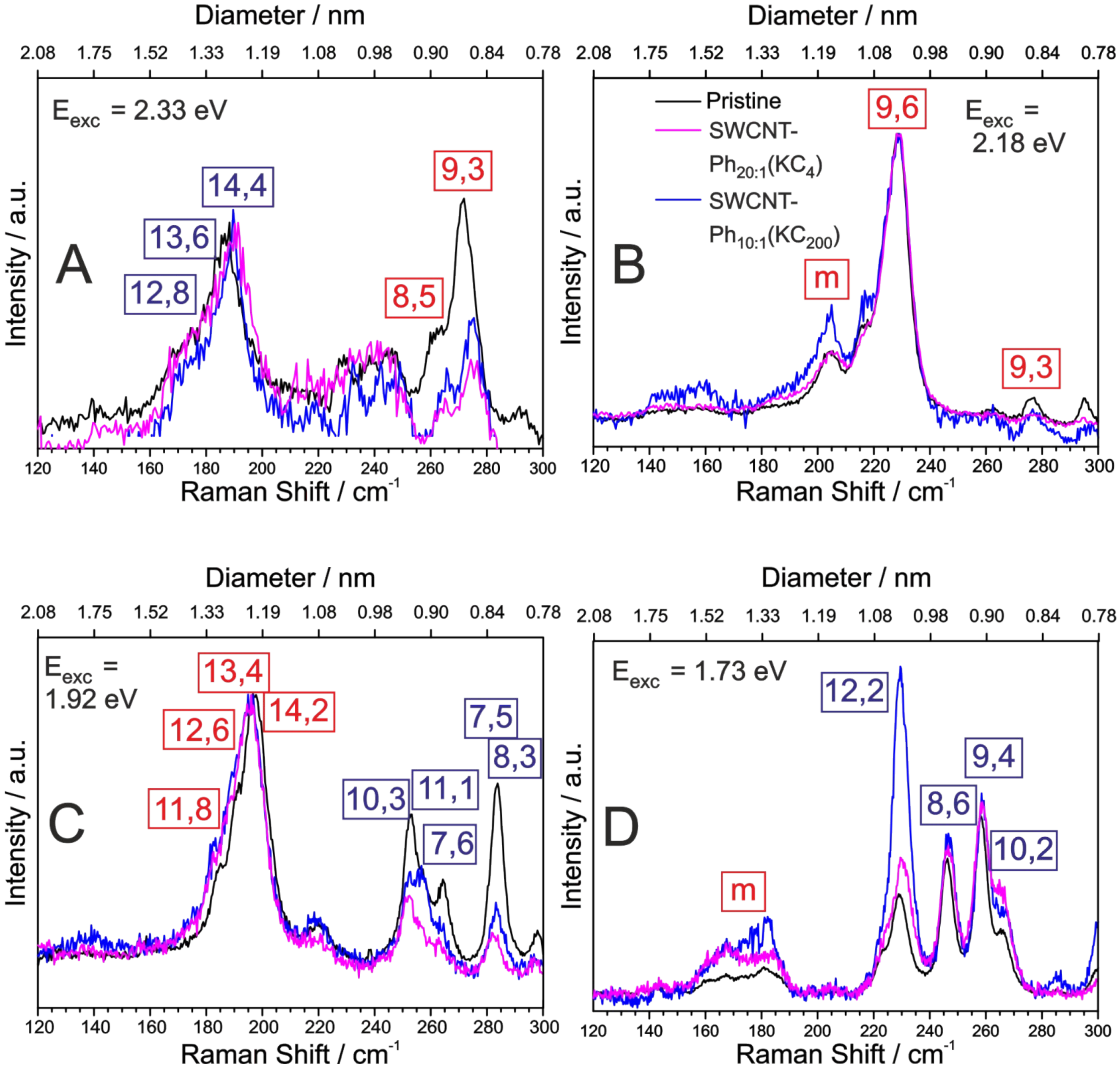

Figure S7: Normalized Raman spectra of the RBM region of pristine HiPco SWCNTs (black) alongside compound SWCNT-$Ph_{20:1}(KC_4)$ (pink) and SWCNT-$Ph_{10:1}(KC_{200})$ (blue) at 2.33 eV (A), 2.18 eV (B), 1.92 eV (C), and 1.73 eV (D) excitation laser energy. Each peak is assigned with nanotube indices (m,n) in red (metallic SWCNTs) or purple (semiconducting SWCNTs, when precise assignment was possible, otherwise with "m" or "sc", respectively.

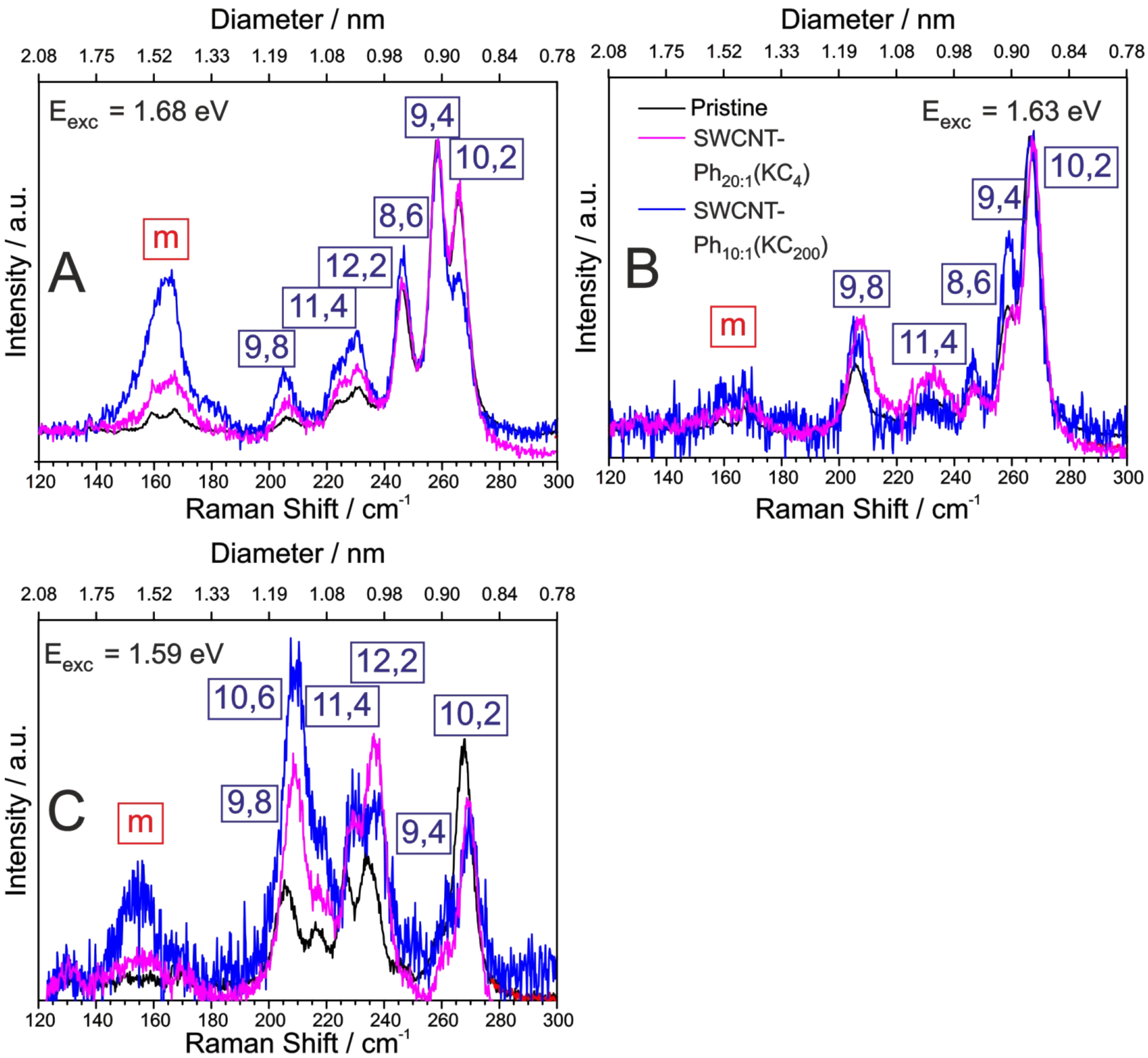

Figure S8: Normalized Raman spectra of the RBM region of pristine HiPco SWCNTs (black) alongside compound SWCNT-Ph$_{20:1}$(KC$_4$) (pink) and SWCNT-Ph$_{10:1}$(KC$_{200}$) (blue) at 1.681 eV (A), 1.63 eV (B), and 1.59 eV (C) excitation laser energy. Each peak is assigned with nanotube indices (m,n) in red (metallic SWCNTs) or purple (semiconducting SWCNTs), when precise assignment was possible, otherwise with “m” or “sc”, respectively.

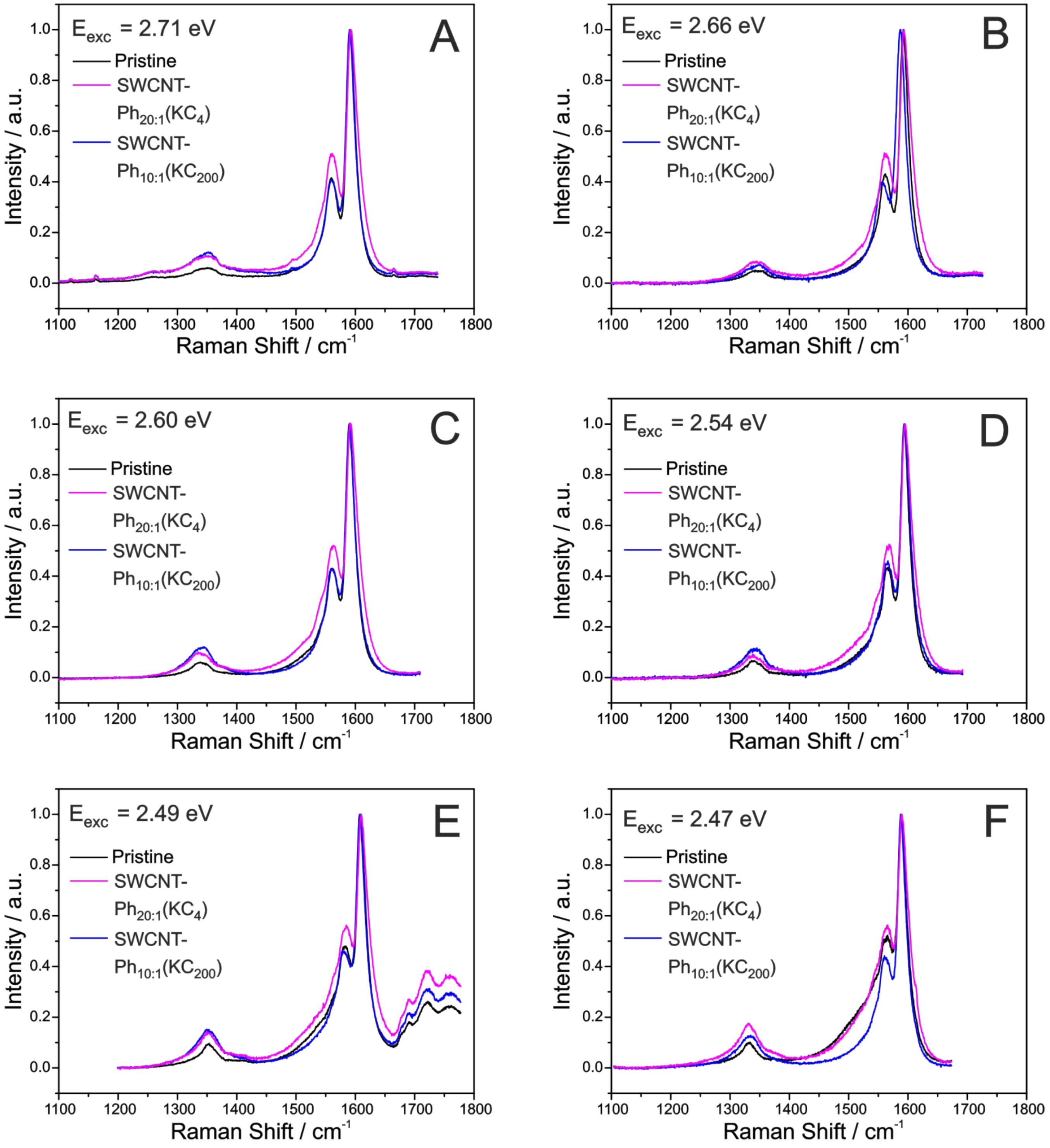

Figure S9: Normalized Raman spectra of the D-band and G-band region of pristine HiPco SWCNTs (black) alongside compounds SWCNT-$Ph_{20:1}(KC_4)$ (pink) and SWCNT-$Ph_{10:1}(KC_{200})$ (blue) at 2.71 eV (A), 2.66 eV (B), 2.60 eV (C), 2.54 eV (D), 2.49 eV (E), and 2.47 eV (F) excitation laser energy.

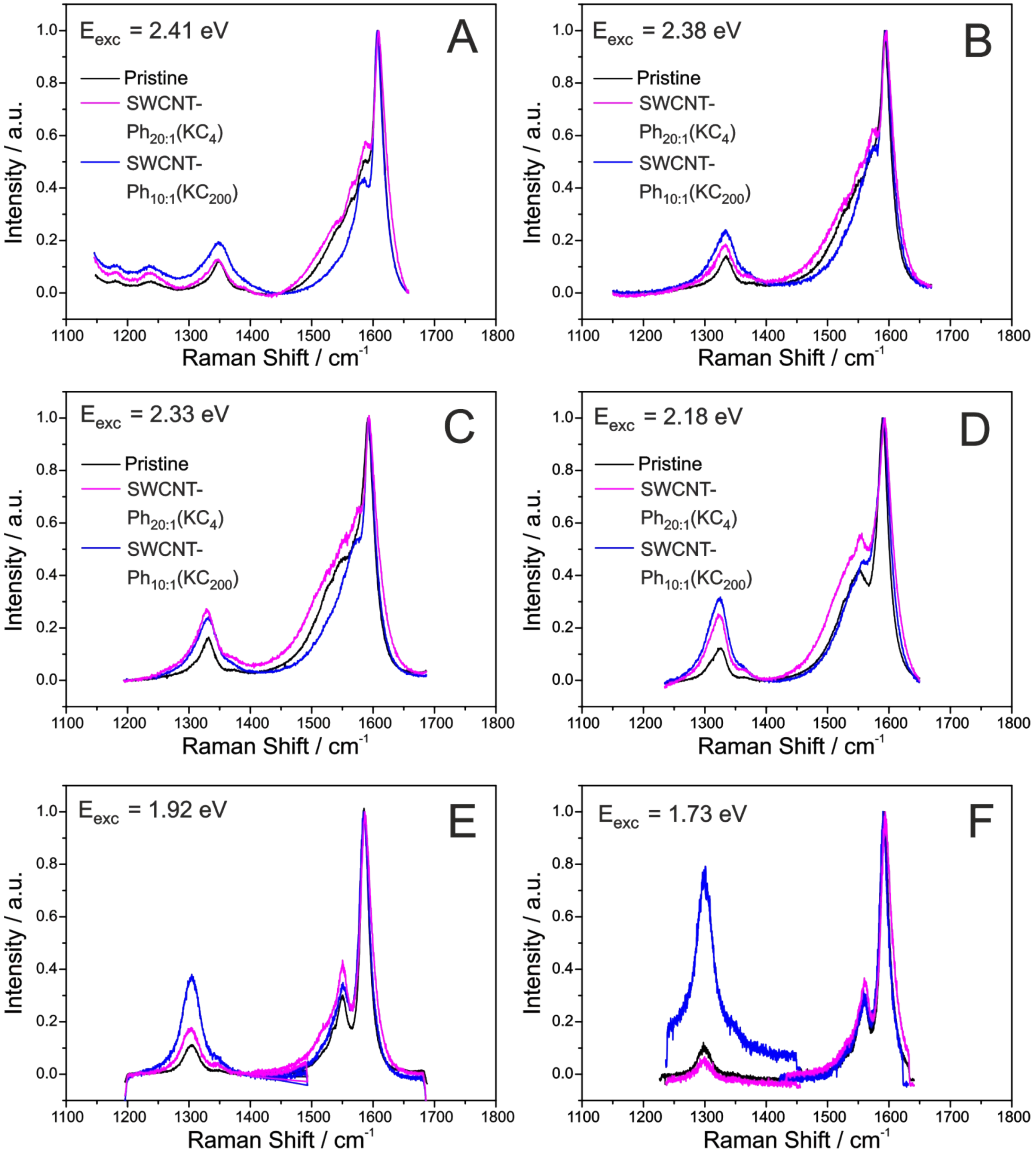

Figure S10: Normalized Raman spectra of the D-band and G-band region of pristine HiPco SWCNTs (black) alongside compounds SWCNT-$Ph_{20:1}(KC_4)$ (pink) and SWCNT-$Ph_{10:1}(KC_{200})$ (blue) at 2.41 eV (A), 2.38 eV (B), 2.33 eV (C), 2.18 eV (D), 1.92 eV (E), and 1.73 eV (F) excitation laser energy.

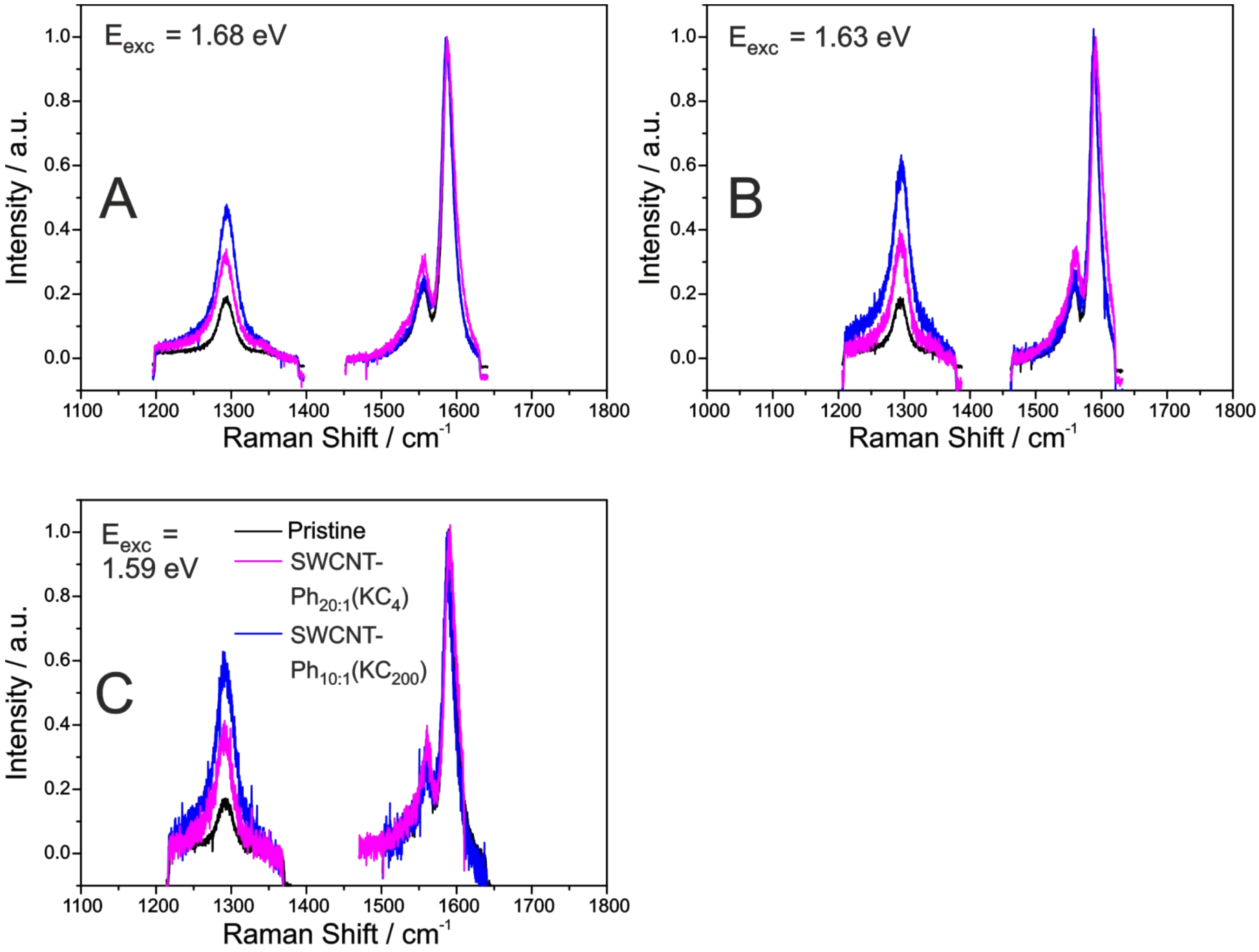

Figure S11: Normalized Raman spectra of the D-band and G-band region of pristine HiPco SWCNTs (black) alongside compounds SWCNT-Ph$_{20:1}$(KC$_4$) (pink) and SWCNT-Ph$_{10:1}$(KC$_{200}$) (blue) at 1.68 eV (A), 1.63 eV (B), and 1.59 eV (C) excitation laser energy.

S6. Supplementary TG-MS and TG-GC-MS Data

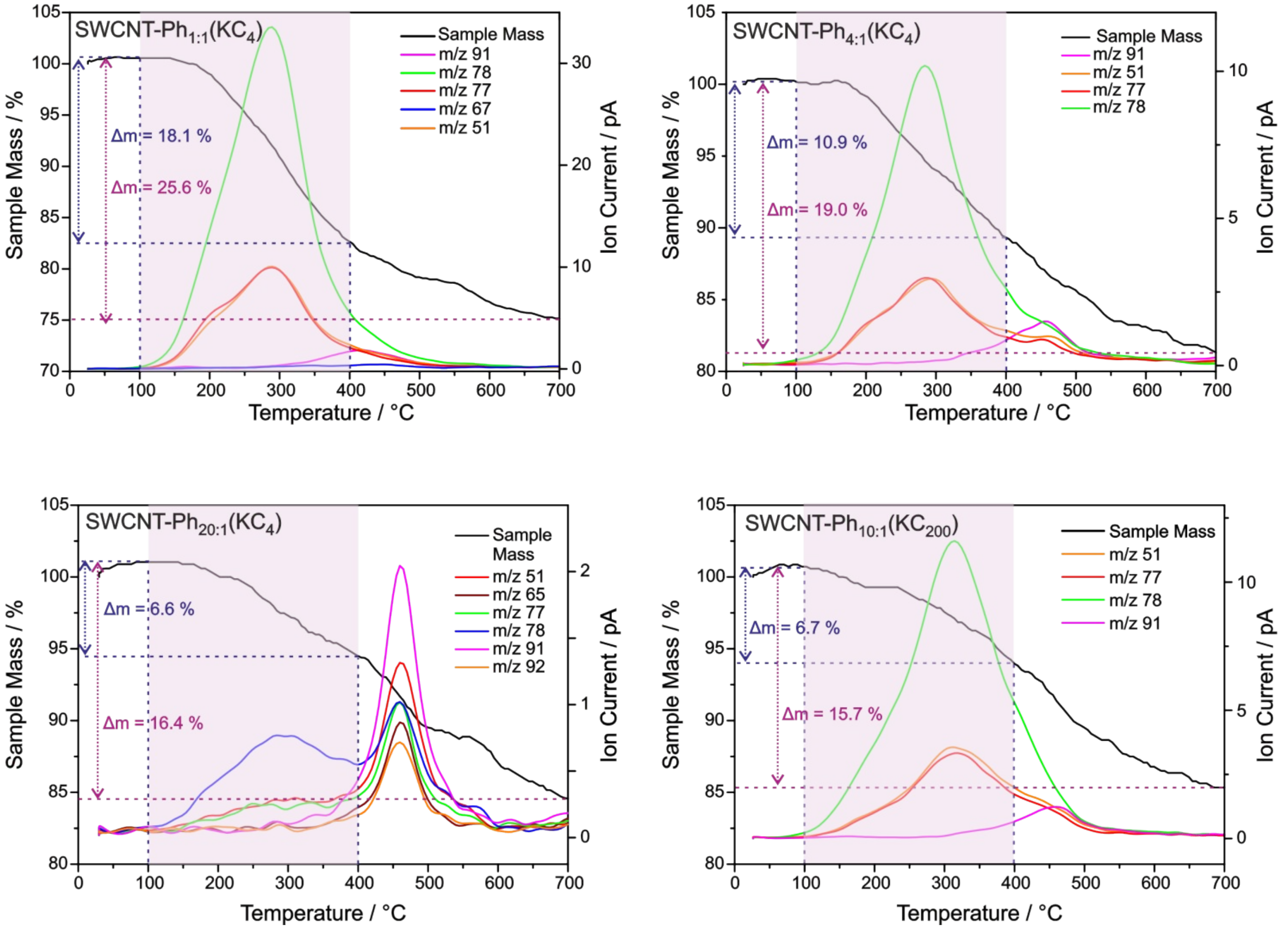

Figure S12: TGA mass loss and MS ion current traces for reductively arylated sample SWCNT-Ph$_{C:Ph}$(KC$_x$)– m/z 78 can be attributed to the protonated detached aryl moieties $C_6H_6^{\cdot +}$ (other fragmentation products are provided in Figure S13).

In order to calculate the degree of functionalization (DoF), several assumptions were made beforehand:

(i) The DoF is defined as the amount of phenyl groups per nanotube carbon and is between 0 and 100 %.

(ii) The mass loss from 100 – 400 °C observed in TGA experiments is caused purely by the loss of covalently sidewall-bonded phenyl groups (m(FG)). This is indicated by TG-MS experiments, where the m/z 78 ion current mainly occurs in this region. Minor amounts of side product as well as adsorbed volatiles like water, gases or even benzene (side product of diazotation) are ignored.

(iii) The mass at 700 °C observed in TGA experiments is remaining pure nanotube carbon (m(C)). This neglects the fact that catalysts (mainly Fe) and other residues like oxygen, hydrogen or amorphous carbon might be present.

Conclusively, the degree of functionalization can be calculated by following formula:

$$DoF = \frac{n(FG)}{n(C)} = \frac{m(FG) * M(C)}{M(FG) * m(C)} \quad (S1),$$

in which

- DoF depicts the degree of functionalization,
- n(FG) and n(C) denote the molar amount of functional group and the molar amount of nanotube carbon, respectively
- m(FG) and m(C) denominate the mass of the functional group and the mass of carbon, respectively, and
- M(FG) and M(C) are the molar mass of the functional group (phenyl, 77.10 g/mol) and the molar mass of carbon (12.01 g/mol), respectively.

Especially (ii) and (iii) are approximations, hence, the degrees of functionalization obtained by this method should always be handled with care and treated as an estimation only.

Table S1: Summary of important Raman and TGA results for all synthesized SWCNT-$Ph_{C:Ph}(KC_x)$ samples. Raman I_D/I_G ratios for 2.33 eV (532 nm) and 1.58 eV (785 nm) laser excitation energy are shown (statistical mean peak intensities determined by SRS). TGA results contain mass of the functional group (m(FG)) and mass of nanotube carbon (m(C)) alongside the calculated degree of functionalization as determined by formula (S1).

Sample	I_D/I_G (2.33)	I_D/I_G (1.58)	m(FG) / %	m(C) / %	Degree of Functionalization / %
SWCNT-$Ph_{1:1}(KC_4)$	0.97	2.35	18.1	74.4	3.8
SWCNT-$Ph_{4:1}(KC_4)$	0.47	0.47	10.9	81.0	2.1
SWCNT-$Ph_{20:1}(KC_4)$	0.17	0.17	6.6	83.6	1.2
SWCNT-$Ph_{10:1}(KC_{200})$	0.39	0.53	6.7	84.3	1.2

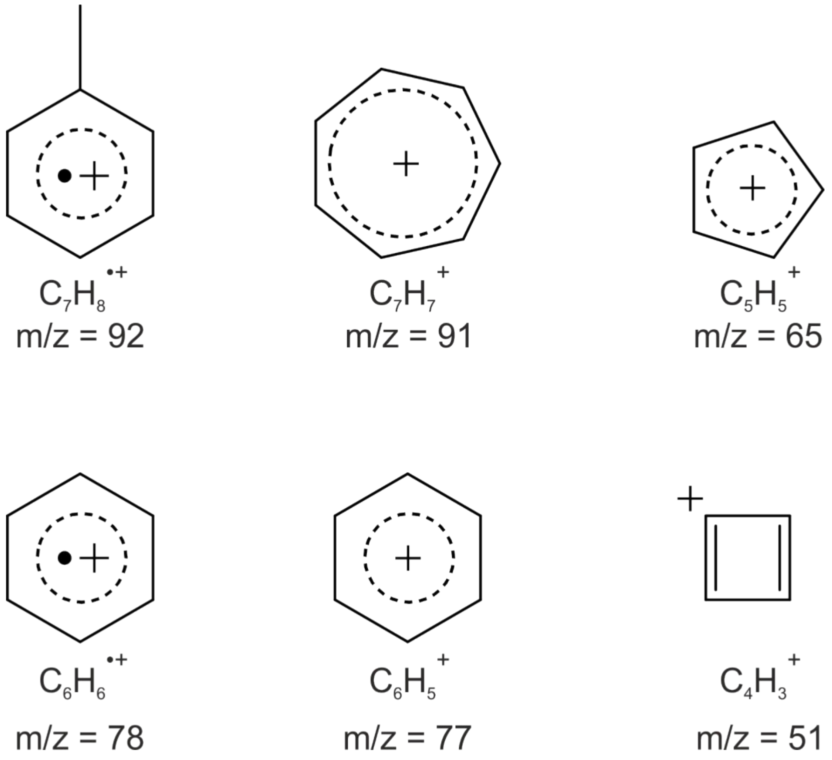

Figure S 13: Cationic fragments of toluene (3 top fragments) and benzene (3 bottom fragments) which are formed during fragmentation in the mass spectrum with corresponding chemic empirical formula and mass-to-charge-ratio.

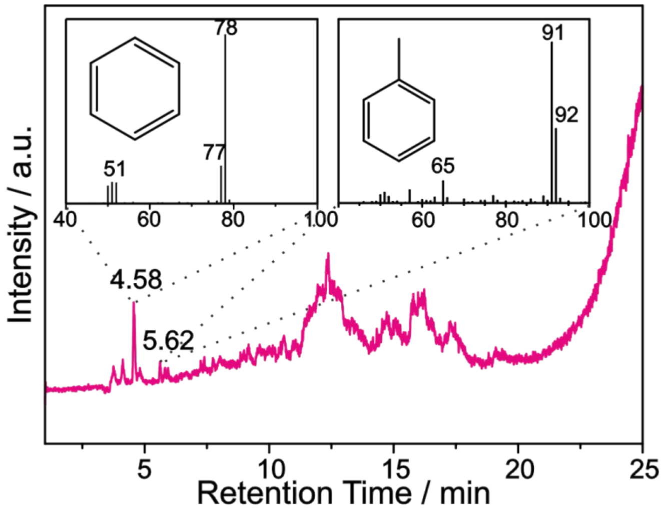

Figure S14:. Gas chromatogram of SWCNT-$Ph_{20:1}(KC_4)$ (pink) at an injection temperature of 480 °C. Inset: MS spectra of the peaks at 4.6 min and 5.6 min retention time, indicative of the thermally detached phenyl and tolyl moieties, respectively, indicating degradation of the SWCNT lattice.[2]

References

1. J. Goclon, M. Kozlowska and P. Rodziewicz, *Chem. Phys. Lett.*, 2014, **598**, 10-16.
2. G. Abellán, M. Schirowski, K. F. Edelthalhammer, M. Fickert, K. Werbach, H. Peterlik, F. Hauke and A. Hirsch, *J. Am. Chem. Soc.*, 2017, **139**, 5175-5182.
